# Sustainable Natural Bio‐Origin Materials for Future Flexible Devices

**DOI:** 10.1002/advs.202200560

**Published:** 2022-03-24

**Authors:** Lingyi Lan, Jianfeng Ping, Jiaqing Xiong, Yibin Ying

**Affiliations:** ^1^ Laboratory of Agricultural Information Intelligent Sensing School of Biosystems Engineering and Food Science Zhejiang University Hangzhou Zhejiang 310058 China; ^2^ Key Laboratory of Intelligent Equipment and Robotics for Agriculture of Zhejiang Province Hangzhou Zhejiang 310058 China; ^3^ Innovation Center for Textile Science and Technology Donghua University 2999 North Renmin Road Shanghai 201620 China

**Keywords:** bio‐origin materials, energy devices, green flexible electronics, sensors, sustainable future

## Abstract

Flexible devices serve as important intelligent interfaces in various applications involving health monitoring, biomedical therapies, and human–machine interfacing. To address the concern of electronic waste caused by the increasing usage of electronic devices based on synthetic polymers, bio‐origin materials that possess environmental benignity as well as sustainability offer new opportunities for constructing flexible electronic devices with higher safety and environmental adaptivity. Herein, the bio‐source and unique molecular structures of various types of natural bio‐origin materials are briefly introduced. Their properties and processing technologies are systematically summarized. Then, the recent progress of these materials for constructing emerging intelligent flexible electronic devices including energy harvesters, energy storage devices, and sensors are introduced. Furthermore, the applications of these flexible electronic devices including biomedical implants, artificial e‐skin, and environmental monitoring are summarized. Finally, future challenges and prospects for developing high‐performance bio‐origin material‐based flexible devices are discussed. This review aims to provide a comprehensive and systematic summary of the latest advances in the natural bio‐origin material‐based flexible devices, which is expected to offer inspirations for exploitation of green flexible electronics, bridging the gap in future human–machine–environment interactions.

## Graphical abstract

This review delivers a concept of sustainable development of future “green” flexible devices based on natural bio‐origin materials. Intrinsically, the flexible devices fabricated with natural bio‐origin materials are desired as intelligent bio‐interfaces to bridge the communication gap for human–machine–environment interactions.

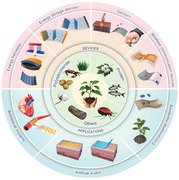

## 1 Introduction

The past few decades have witnessed the fast‐growing development of electronic devices.^[^ ^1^ ^]^ The rapid advancement of electronic technology has changed our daily lives significantly.^[^ ^2^ ^]^ Particularly, flexible electronics that can be conformally attached to curved and dynamic surfaces have been extensively studied,^[^ ^3^ ^]^ which can eliminate the discomfort of users and thus allowing long‐term on‐skin applications for energy management, health‐monitoring and information communication.^[^ ^4^ ^]^ Over the past five years, flexible electronics has experienced rapid growth due to the advances in materials^[^ ^5^ ^]^ and mechanics designs.^[^ ^6^ ^]^ In the foreseeable future, flexible electronics are expected to take more important roles as intelligent bio‐interfaces in healthcare,^[^ ^7^ ^]^ biomedical therapy,^[^ ^8^ ^]^ and human–machine–environment interface.^[^ ^9^ ^]^

Although the rapid development of flexible electronics brings convenience to our lives, speedy up‐gradation along with the rapid proliferation of these devices is giving rise to an increased amount of electronic waste (E‐waste) around the world.^[^ ^10^ ^]^ Currently, the E‐waste produced every year significantly increases the demand for landfill space and causes environmental burdens at the same time.^[^ ^11^ ^]^ Recycling and disposal of E‐waste are considered as a global problem, which poses great demand for developing sustainable systems.^[^ ^12^ ^]^ The E‐waste problem can be partially ascribed to the used non‐degradable synthetic polymers for electronic devices fabrication. Therefore, given the severity of the E‐waste problem, there is an urgent need for developing environmentally‐friendly materials for constructing newly flexible electronics that are eco‐friendly, disposable, and biodegradable. Recently, increasing attention has been paid on searching more sustainable materials as components of flexible electronics.^[^ ^13^ ^]^

In contrast to synthetic polymers, natural bio‐origin materials have been considered as promising building blocks for constructing next‐generation electronics due to their unique properties.^[^ ^14^ ^]^ Typically, natural bio‐origin materials are naturally occur within different kinds of organisms such as microorganisms,^[^ ^15^ ^]^ animals, and plants. Natural bio‐origin materials offer appealing properties including environmental benignity, natural abundance, low cost, biodegradability, biocompatibility, and less energy consumption during their processing in contrast to most synthetic materials.^[^ ^16^ ^]^ To date, many studies have researched applications of different types of bio‐origin materials such as polysaccharides like cellulose,^[^ ^17^ ^]^ starch,^[^ ^18^ ^]^ chitin/chitosan,^[^ ^19^ ^]^ and proteins like silk fibroin,^[^ ^20^ ^]^ collagen,^[^ ^21^ ^]^ and gelatin^[^ ^22^ ^]^ in flexible electronics. The use of these sustainable bio‐origin materials offers numerous merits in environmental conservation in comparison to the traditional non‐biodegradable synthetic materials.^[^ ^23^ ^]^ Besides, natural bio‐origin materials also offer other advantages such as hierarchical structure,^[^ ^24^ ^]^ morphological diversity,^[^ ^25^ ^]^ and numerous functional groups for modifications, making them attractive for constructing different kinds of flexible electronics.^[^ ^26^ ^]^

**Figure 1:**
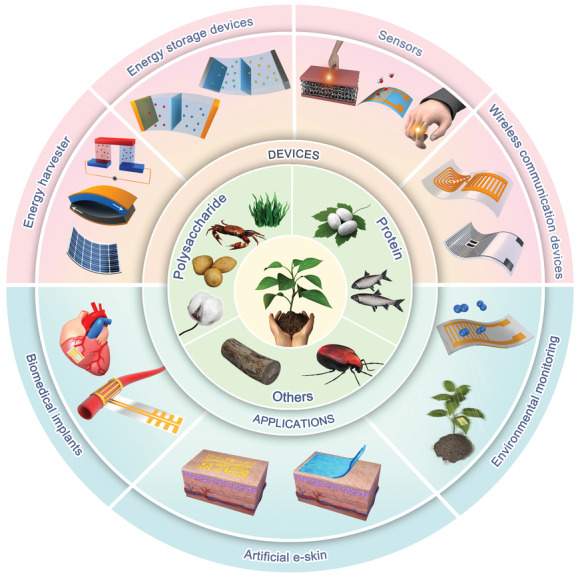
Schematic illustration showing the frame of this review. It delivers a concept of sustainable development of future “green” flexible devices based on natural bio‐origin materials. Intrinsically, the flexible devices are desired as intelligent bio‐interfaces to bridge the communication gap for human‐machine‐environment interactions. Natural bio‐origin materials with merits such as low cost, eco‐friendliness, biocompatibility, and recyclability, enabling devices with desired sustainability for safe and long‐term interactions with objects and environment around.

In this comprehensive review, we summarized the recent progress on flexible devices that are partially or fully fabricated with natural bio‐origin materials. The scope of the review is outlined in **Figure** **1**, which presents a sustainable concept of future “green” flexible devices based on natural bio‐origin materials. Firstly, we highlight the sources and chemical structures of various natural bio‐origin materials, which are classified into polysaccharides (cellulose, starch, chitin‐chitosan, and alginate), proteins (silk fibroin, collagen, and gelatin), and other types such as lignin and shellac. Understanding the fundamental structures and characteristics of these bio‐origin materials is vital for designing and manufacturing devices for emerging applications. Then, we summarized the functions and applications of the bio‐origin materials for fabricating different kinds of flexible devices. The devices are sorted into four categories based on their functions. That is, energy harvesters that could harvest various kinds of energy such as mechanical deformation/vibration (triboelectric and piezoelectric nanogenerator), heat (thermoelectric generator), and solar radiations (solar cell) from the environment and convert them into electricity; energy storage devices (supercapacitor and battery) that could power portable devices; sensors that transduce changes such as pressure, strain and humidity into measurable signals in different conditions; signal transmission devices such as radio‐frequency identification device (RFID) and near‐field communication (NFC) for wireless communication between different devices. We believe that the four kinds of devices mentioned above are vital components for building an integral system. The design and functionality of these devices fabricated with the bio‐origin materials as substrate/matrix or active components are introduced scrupulously. Following this, the applications of these bio‐origin material‐based devices are discussed, which mainly focus on three applications: biomedical implants, artificial electronic skin (e‐skin), and environmental monitoring. Finally, we discuss the challenges and outlooks for the bio‐origin material‐based devices. By leveraging the distinct merits of bio‐origin materials, possible strategies for designing and constructing next‐generation flexible devices are provided. By this review, we want to deliver a concept that natural bio‐origin material‐based flexible devices would play a vital role in building intelligent bio‐interfaces in future applications to meet the requirement for sustainable development. The aim of this review is not only to highlight materials and devices fabrication but also to expose roadmaps to be followed that can ultimately lead to the development of high‐performance bio‐origin material‐based flexible devices for a sustainable future.

## 2 Structures and Fundamental Properties of Natural Bio‐Origin Materials

Nature has provided us with different kinds of natural materials that possess merits of eco‐friendliness, easy processability, and low cost. In this chapter, we overview the structures and fundamental characteristics of natural bio‐origin materials including polysaccharides, proteins, and other natural polymers. Understanding the fundamental structures and characteristics of these bio‐origin materials is vital for designing and manufacturing functional devices for emerging applications. We also discuss the pros and cons of the bio‐origin materials to be used for fabricating flexible devices.

### 2.1 Polysaccharides

Polysaccharides are formed by homopolymers or copolymers of monosaccharides. It is a polymer consist of glucose units linked together by glycosidic bonds in the linear or branched configuration. In nature, there are various kinds of polysaccharides including cellulose, starch, chitin/chitosan, and alginate derived from different sources. The structures and features of these polysaccharides are introduced in detail in the following part.

#### 2.1.1 Cellulose

**Figure 2:**
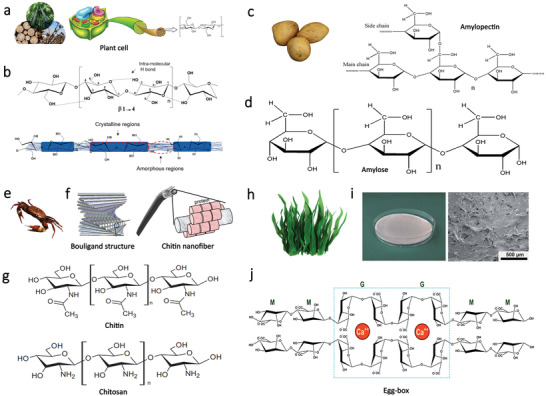
Schematic illustration of structures and morphologies of different kinds of polysaccharides. a) Representative source of cellulose and its hierarchical and chemical structure. Reproduced with permission.^[^ ^40^ ^]^ Copyright 2014, MDPI. b) Cellulose consists of repeating unit with the *β*‐(1,4)‐glycosidic linkage and the crystalline and disordered regions. Reproduced with permission.^[^ ^41^ ^]^ Copyright 2019, MDPI. c) Typical source of starch. d) Chemical structure of amylose and amylopectin. e) Typical source of chitin and chitosan. f) Schematic presentation of the exoskeleton structure of chitin. Reproduced with permission.^[^ ^42^ ^]^ Copyright 2012, The Royal Society of Chemistry. g) Chemical structure of chitin and chitosan. Reproduced with permission.^[^ ^43^ ^]^ Copyright 2013, Elsevier. h) Typical source of alginate. (i) Photo and surface SEM image of the calcium alginate film. Reproduced with permission.^[^ ^44^ ^]^ Copyright 2018, The Royal Society of Chemistry. j) Scheme of “Egg‐box” structure in alginate hydrogel crosslinked with calcium ions. Reproduced with permission.^[^ ^45^ ^]^ Copyright 2019, Springer Nature.

Cellulose is a natural fibrous polymer that is highly abundant, low cost, and renewable on earth.^[^ ^27^ ^]^ As illustrated in **Figure** **2**a, it is the main component of plant cell walls. Typically, cellulose is extracted from natural plants including wood,^[^ ^28^ ^]^ cotton,^[^ ^29^ ^]^ and bamboo.^[^ ^30^ ^]^ Besides, fungi and bacteria also possess the ability to produce cellulose, namely, bacterial cellulose (BC).^[^ ^31^ ^]^ Chemically, cellulose with a general formula of (C_6_H_10_O_5_)*_n_* comprises of repeating linear chains of *β*­d­glucopyranose monomers covalently linked via *β*­1,4 glycosidic bonds. As shown in Figure 2b, intramolecular hydrogen bonding can be formed between oxygen and hydrogen atoms of the adjacent rings, leading to more stable glycosidic linkage motifs as well as linear‐chain configuration. Besides, inter‐chain hydrogen bonds along with van der Waals forces can occur between the neighboring polymer chains, which promote their parallel stacking and can be assembled into elementary fibrils. These elementary fibrils can form microfibers by assembling into rectangular arrays. Two regions, that is, crystalline (highly ordered) and amorphous (disordered) structures can be found within these cellulose fibers. The distribution of these two regions usually relies on raw materials and pretreating techniques. By using different treating methods, cellulose fibers can be processed into various morphologies such as cellulose nanofibril (CNF), and cellulose nanocrystal (CNC). Noted that BC is naturally synthesized by some bacterial via a bottom‐up procedure, which is different from the methods for CNC and CNF preparation. CNC is rigid nanorod that is relevant to the crystalline regions of cellulose fibers, which can be obtained through a mild acid hydrolysis.^[^ ^32^ ^]^ During the process, acid would infiltrate into the amorphous regions of the cellulose fibers and induce the cleavage of glycoside bonds, which leads to the dissolution of amorphous parts and the crystalline CNC is obtained. Even though CNC can be processed into transparent films for different applications, the CNC films are usually brittle due to the crystalline nature of CNC. Unlike CNC, CNF that consist of both crystalline and amorphous domains are more flexible due to the higher aspect ratio.^[^ ^33^ ^]^ Generally, CNF can be produced by mechanical exfoliation of cellulose fiber under a high shear force. Before separating the stacked fibrils, certain treatments such as alkaline,^[^ ^34^ ^]^ radiant,^[^ ^35^ ^]^ chemical,^[^ ^36^ ^]^ and enzymatic^[^ ^37^ ^]^ are required to facilitate the fibrillation of cellulose fibers. These pretreatment procedures are vital for reducing energy consumption, which allows the industrialization of CNF. The CNF with a high aspect ratio is abundant in hydroxyl groups, forming strong hydrogen bonds between each nanofiber, which endows CNF with unique properties including improved mechanical strength as well as gel‐like feature.^[^ ^38^ ^]^ In addition to preparing nanoscale structures of cellulose, chemical modification of cellulose fibers is also extensively studied to meet different practical applications. By esterification or etherification of the hydroxyl groups, a variety of derivatives including methylcellulose (MC), ethyl cellulose (EC), hydroxyethyl cellulose (HEC), cellulose acetate (CA) can be produced, which are more facilely processable and widely applicable in different fields.^[^ ^39^ ^]^

#### 2.1.2 Starch

Starch is a biodegradable and renewable polymer that is abundantly available in nature. It can be extracted from various low‐cost sources such as corn, wheat, and potato (Figure 2c).^[^ ^46^ ^]^ Starch is semi‐crystalline, and its structure consists of glucose units linked via glycosidic bonds. Typically, there are two types of molecules in most starches: amylose and amylopectin. As shown in Figure 2d, amylose is amorphous with a linear (1,4)‐linked *α*‐glucopyranose, while amylopectin is crystalline and it is a branched molecule of (1,4)‐linked *α*‐glucopyranose with *α*‐(1,6) branch linkages.^[^ ^47^ ^]^ The different chemical structures of the two types of molecules lead to different physical properties, enzyme hydrolysis susceptibility, as well as water solubility.^[^ ^48^ ^]^

Generally, starch is difficult to dissolve and process because the hydroxyl groups on the starch polymer chains could form strong intermolecular or intramolecular hydrogen bonds.^[^ ^49^ ^]^ Instead of dissolution, starch granules would integrate with water molecules through hydrogen bonding between the starch chains, tuning into hydrated starch. After heating process, starch gel can be formed, this process is called gelatinization.^[^ ^50^ ^]^ However, films obtained from native starch usually exhibit weak mechanical strength as well as poor flexibility due to the massive hydrogen bonding within the starch chains. As such, chemical or physical modifications of native starch are extensively explored to overcome these limitations.^[^ ^51^ ^]^ Through chemical reactions such as cross‐linking or replacement by other molecules,^[^ ^52^ ^]^ the modified starch with enhanced properties shows wider applications in constructing flexible devices.

#### 2.1.3 Chitin and Chitosan

Chitin is a nitrogenous and fibrous polysaccharide that is abundant in nature, which is a vital constituent of the cell wall of fungi. It also acts as the exoskeleton of insects as well as arthropods, which plays an important role in maintaining cell integrity.^[^ ^53^ ^]^ Commercially, chitin can be produced massively from crab and shrimp shell wastes (Figure 2e).^[^ ^54^ ^]^ As illustrated in Figure 2f, the chitin naturally occurs as supramolecular crystalline nanofibers which are wrapped by protein layers. Structurally, it is composed of repeating units of N‐acetyl glucosamine linked with *β* (1‐4) glycosidic bonds.^[^ ^42^, ^55^ ^]^ Chitin is insoluble in water due to intramolecular and intermolecular hydrogen bonds with adjacent —NH or —OH functions groups.^[^ ^19^ ^]^ Chitosan is the deacetylated form of chitin (Figure 2g).^[^ ^56^ ^]^ The acetylated amine groups in chitin and primary aliphatic amine groups in chitosan make them more suitable for certain reactions of amines.^[^ ^57^ ^]^ Nevertheless, compared with chitin, chitosan is more chemically active because of the primary and secondary hydroxyl groups on each repeat unit.^[^ ^43^ ^]^ These groups provide an excellent platform for chemical modifications to tune either the mechanical or physical characteristics of chitosan.^[^ ^58^ ^]^ Chitin and chitosan with biocompatibility, biodegradability, bioactivity, and non‐toxicity are both considered to be suitable and essential biomaterials for extensive applications.^[^ ^59^ ^]^ By bottom‐up self‐assembly of chitin nanofiber, flexible and transparent films with smooth surface can be obtained, showing great futuristic potential in fabricating flexible and biofriendly devices.^[^ ^55^ ^]^

#### 2.1.4 Alginate

Alginate is a hydrophilic and anionic polysaccharide that can be extracted from brown sea algae (Figure 2h).^[^ ^44^ ^]^ It can be processed into a free‐standing film via drop‐casting (Figure 2i). As a biomaterial, it is extensively used as the supporting matrix for tissue repair or regeneration.^[^ ^60^ ^]^ Structurally, alginate is a linear copolymer composed of alternating blocks of (1,4)‐*α*‐l‐guluronate (G unit) and (1,4)‐*β*‐d‐mannuronate (M unit).^[^ ^61^ ^]^ As a naturally formed polysaccharide, alginate shows a pH‐dependent anionic nature and can interact with cationic polyelectrolytes and proteoglycans.^[^ ^62^ ^]^ In the presence of calcium ions, the carboxylic acid groups of alginate in G units of adjoining polymer chains crosslink would interact with Ca^2+^, forming calcium alginate that displays egg‐box model with excellent mechanical properties, as illustrated in Figure 2j.^[^ ^45^, ^63^ ^]^ By this process, alginate hydrogels can be obtained from aqueous solutions, which show wide applications in tissue engineering and drug delivery.^[^ ^64^ ^]^

### 2.2 Protein

#### 2.2.1 Silk Fibroin

**Figure 3:**
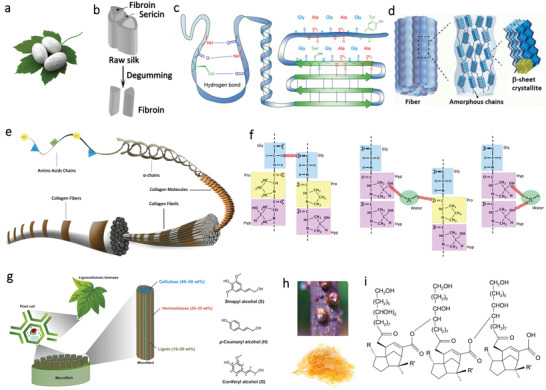
Schematic illustration of structures and morphologies of different kinds of protein. a) Schematic illustration of Bombyx mori silk cocoons. b) The structure of raw silk. Reproduced with permission.^[^ ^67^ ^]^ Copyright 2015, Elsevier. c) Schematic of SF molecular chain showing the hydrogen bonding and secondary structures. Reproduced with permission.^[^ ^71b^ ^]^ Copyright 2019, National Academy of Sciences, USA. d) Fibroin assembled from nanofibril units. Reproduced with permission.^[^ ^73^ ^]^ Copyright 2015, Wiley‐VCH. e) Sketch of the four‐level structure of collagen fiber. Reproduced with permission.^[^ ^76^ ^]^ Copyright 2019, Wiley‐VCH. f) The primary structure of gelatin. Reproduced with permission.^[^ ^22^ ^]^ Copyright 2015, Elsevier. g) Structure of lignocellulosic biomass and monolignol monomer species of lignin. Reproduced with permission.^[^ ^77^ ^]^ Copyright 2020, Cell Press. h) Photograph of the female lac inset and shellac. Reproduced with permission.^[^ ^78^ ^]^ Copyright 2017, Springer. i) Chemical structure of shellac. Reproduced with permission.^[^ ^79^ ^]^ Copyright 2017, The Royal Society of Chemistry.

Natural silk fiber is extensively employed for fabricating flexible devices due to its excellent mechanical performance.^[^ ^65^ ^]^ Typically, it can be produced by arthropods like silkworms, spiders, and scorpion. Among them, bombyx mori (B. mori) silkworms are considered as major producer of silk during the metamorphosis process (**Figure** **3**a).^[^ ^66^ ^]^ Silk fiber consists of a central protein known as fibroin encased with a layer of another protein called sericin, as illustrated in Figure 3b. Fibroin takes possession of the major components of more than 70% weight of silk fiber.^[^ ^67^ ^]^ Sericin is responsible to glue the fibroin fibers into a cocoon.^[^ ^68^ ^]^ It is usually removed during the degumming process as it may cause immune responses to humans.^[^ ^69^ ^]^ After removing sericin, the obtained silk fibroin (SF) can be dissolved in an aqueous solution.

SF with a diameter of about 10–25 µm is consists of two proteins: a light (L) chain polypeptide and a heavy (H) chain polypeptide with a ratio of 1:1. The two polypeptides are linked together via a disulfide bond.^[^ ^70^ ^]^ The H‐chains could form discrete‐sheet crystallites and serve as the major structural constituent, while L‐chain with a much smaller size plays little mechanical role (Figure 3c).^[^ ^71^ ^]^ Typically, SF is considered as a natural amphiphilic block copolymer that consists of hydrophobic and hydrophilic blocks, giving rise to both the flexibility and toughness of SF.^[^ ^72^ ^]^ As shown in Figure 3d, at microscopic level, crystalline domains are highly oriented, which act as crosslinking points in the amorphous matrix.^[^ ^73^ ^]^ Over the past years, SF shows great potential in biomedical applications due to the favorable biocompatibility and controllable degradability.^[^ ^74^ ^]^ Moreover, SF with excellent mechanical strength as well as outstanding flexibility making it one of the most promising natural protein fibers in the applications for flexible and green devices.^[^ ^75^ ^]^

#### 2.2.2 Collagen

Collagen is an insoluble fibrous protein in human bodies that are responsible for supporting tissues including tendons, skin, and teeth.^[^ ^21^ ^]^ It can be obtained from animals such as bovine, porcine, and marine organisms such as scale fish and fish skin.^[^ ^80^ ^]^ Collagen possesses a complicated hierarchical structure with four levels (Figure 3e).^[^ ^76^ ^]^ The primary structure is amino acid triplet chains and the repeated chains form the secondary structure. The tertiary structure is a triple helix, which is known as the *α*‐chain that is composed of more than 1000 amino acids.^[^ ^81^ ^]^ By connecting three chains via interchain hydrogen bonds, a triple‐helical region can be formed. The unique triple helix structure makes its molecular structure very stable.^[^ ^82^ ^]^ The quaternary structure is formed by self‐assembly, which is called collagen fibrils or collagen fibers. Based on the assembly way of polypeptide chains, different lengths of helix structures, and variations in termination of helix, different classification of collagen can be identified.^[^ ^82^ ^]^ Typically, collagen cannot be dissolved in water or saline solutions, but it is soluble in acidic solution with a pH range of 2–4.^[^ ^21^ ^]^ A transparent collagen film can be formed by acid‐soluble collagen, which can serve as substrates for fabricating flexible devices.^[^ ^83^ ^]^ Furthermore, due to its high mechanical strength and biodegradability, collagen also holds great promise as a biomaterial in biomedical applications.^[^ ^84^ ^]^

#### 2.2.3 Gelatin

Gelatin is a translucent protein compound obtained by partially hydrolyzed and denatured of collagen. As shown in Figure 3f, the primary structure of gelatin is the same for all types, which consists of eighteen groups of amino acids.^[^ ^22^ ^]^ There is only a small difference in the component ratio owing to different source of raw materials in combination with the pretreatment and extraction method.^[^ ^85^ ^]^ Nevertheless, the secondary structure of gelatin is formed by various polypeptide chains including *α*‐chains, *β* chains, and *γ* chains.^[^ ^86^ ^]^ Unlike native collagen, Gelatin is soluble in water because its triple‐helical peptides structure would “unwind” into single peptide chains.^[^ ^22^ ^]^ So far, gelatin has shown wide potential applications in various fields because of its large abundance and biocompatibility.^[^ ^87^ ^]^ However, the hygroscopic nature of gelatin films makes it difficult to be used in high moisture conditions.^[^ ^88^ ^]^ As such, great efforts have been made to develop gelatin‐based films with improved mechanical and water resistance properties by combining with other biopolymers, making gelatin more promising in constructing flexible and robust devices.^[^ ^89^ ^]^

### 2.3 Others

#### 2.3.1 Lignin

Lignin is an amorphous and heterogeneous polymer that is a constituent of lignocellulosic plants' natural fibers along with cellulose and hemicellulose.^[^ ^90^ ^]^ The schematic illustration of the cell wall structure of a lignocellulosic natural plant is shown in Figure 3g, where cellulose is glued by lignin and hemicellulose. Lignin is a macromolecular material consists of three different phenolic alcohol units including p‐coumaryl, coniferyl, and sinapyl monolignols that are linked together by C—C or C—O bond.^[^ ^77^, ^91^ ^]^ The hydrophobicity of lignin makes the cell wall impermeable to water, which could ensure effective water and nutrition transportation in the cells.^[^ ^92^ ^]^ The unique structure of lignin endows it with numerous advantages. For instance, the functional groups such as phenolic units, ketones that can absorb UV make lignin a natural broad‐spectrum sun blocker.^[^ ^93^ ^]^ Besides, the phenolic groups with free radical scavenging capability provide lignin with superior antioxidant properties.^[^ ^94^ ^]^ Moreover, lignin exhibits great thermal stability, which allows it easily to be processed.^[^ ^95^ ^]^

#### 2.3.2 Shellac

Shellac is a natural and biodegradable resin that originated from the secretion of lac insect,^[^ ^96^ ^]^ which is a mixture of polar and non‐polar constituents including aliphatic, shellac, and jalaric alicyclic hydroxy acids, along with polyhydroxy polycarboxylic esters.^[^ ^97^ ^]^ Shellac can be made by rinsing and filtration of crude shellac which is directly secreted by lac bug, the color of shellac varies from light yellow to dark red relying on the types of raw materials (Figure 3h). The chemical structure of shellac is given in Figure 3i.^[^ ^79^ ^]^ Shellac can be widely used in various fields. In the food industry, it is usually used for sealing and glossing due to its ability to form a natural superficial layer that can protect food.^[^ ^98^ ^]^ In the pharmaceutical industry, shellac is often used as taste masking for bitter pills as an alternative to synthetic polymers.^[^ ^99^ ^]^ Shellac is not water‐soluble but can be dissolved in alcohol or alkaline solutions. By drop‐casting shellac solution with ethanol or methanol, a shellac thin film with low surface roughness can be easily fabricated. The shellac thin‐film possesses a dielectric constant between 3–5, making it a great choice for fabricating different kinds of electronics.^[^ ^100^ ^]^ However, it should be note that shellac is a rigid biopolymer, so it is important to obtain flexible formulations for its broader applications. Currently, a formulation is made commercially available and is traded under the name SWANLAC by the company A.F. Sutter. & Co Ltd. In addition, there have been several reports on improving the flexibility of shellac films by adding polymers such as polyethylene glycol (PEG)^[^ ^101^ ^]^ as the plasticizer.

### 2.4 The Pros and Cons of Natural Bio‐Origin Materials

Natural bio‐origin materials possess excellent features such as tailorable chemical composition as well as mechanical properties. Apart from the structural features, they also exhibit great biological characteristics such as abundant supply, biodegradability, biocompatibility, and anti‐microbial activity. The unique structures and characteristics of natural bio‐origin materials make them attractive for the fabrication of sustainable flexible electronic devices. However, the application of natural bio‐origin materials for fabricating electronic devices still shows limitations due to their relatively poor electrical performance in comparison to the conventional electronic materials. To overcome this, dual functional bio‐composites can be prepared by mixing natural bio‐origin materials with conductive materials, enabling an eco‐friendly matrix for protection of the conductive components. The bio‐composites are expected to possess both environmental friendliness as well as high conductivity, which broadens their applications for fabricating electronic devices.

Besides, when serving as the substrate or encapsulation component for electronic devices, the natural bio‐origin materials can be easily dissolved in water/biofluids due to its biodegradability, which may cause the electrical discontinuity or failures of functional devices, leading to difficulties in clinical applications. Therefore, encapsulation layers are required to protect the bio‐origin materials‐based electronic devices from direct contact with environmental elements and maintaining mechanical/electrical robustness, aiming to elongate the lifetime of these electronic devices during the operation. Furthermore, the encapsulation layers with environment‐adaptivity are expected to regulate the deactivation or degradation of the electronic devices precisely with their designed dissolution characteristics. Both inorganic dielectric materials and polymeric substrate materials can be used for encapsulating purposes. For implantable devices, additional biocompatibility and suitable surface adhesion are desired on the encapsulation layers. Synthetic polymers such as polyvinyl alcohol (PVA) and poly(lactic‐*co*‐glycolic acid) (PLGA) are widely used as encapsulating materials. Natural bio‐origin materials like silk and polyanhydrides are also commonly used for fabricating encapsulation layers. The biocompatible materials with variant environmental stability and interface adaptivity provide options for encapsulation in different applications. Moreover, innovation of architectures and environment‐adaptivity of bio‐origin materials is a promising strategy to realize smart encapsulation layers to control the lifetime of devices, catering multi‐scenarios applications.

## 3 Flexible Devices Based on Natural Bio‐Origin Materials

Based on their structures and features, natural bio‐origin materials are attractive building blocks for the fabrication of sustainable flexible devices. Based on the processing methods, natural bio‐origin materials can be processed into diverse regenerated formats. In this chapter, we discuss the different role of natural bio‐origin materials in fabricating different kinds of flexible devices. The devices are sorted into four categories based on their functions. That is, energy harvesters that could harvest various kinds of energy, energy storage devices that could power portable devices, sensors that could transduce changes into measurable signals, and signal transmission devices for wireless communication between different devices.

### 3.1 Energy Harvester

#### 3.1.1 Triboelectric Nanogenerator

To meet the fast‐growing requirements for sustainable power sources, scientists are paying attention to develop technologies that can harvest environmental energy and convert it into electricity. Triboelectric nanogenerator (TENG) is considered as an emerging technology to convert ubiquitous mechanical energy into electricity.^[^ ^102^ ^]^ The working mechanism of TENG involves the coupling of triboelectrification and electrostatic induction.^[^ ^103^ ^]^ The TENG shows merits such as stable output performance, high energy conversion efficiency, and the ability to harvest random or low magnitude of mechanical energy,^[^ ^104^ ^]^ which allows its wide applications in harvesting different kinds of mechanical energy.^[^ ^105^ ^]^ Particularly, recent research in “green” TENG fabricated with biocompatible materials has become a hot topic, aiming to develop biocompatible power source without producing any harmful substances, which can minimize the potential health risks caused by hazardous wastes.^[^ ^106^ ^]^ Compared with synthetic biocompatible polymers, natural bio‐origin materials with large abundance and low cost have been widely employed for the fabrication of TENG (**Table** **1**). The latest research of bio‐origin material‐enabled TENG is summarized as follows.

**Table 1:** Summary of TENG based on natural bio‐origin materials

Natural materials	Functions of the material	Electrode	Output voltage	Output current	Power density	Ref.
Cellulose	Triboelectric layer	Ag nanowires	21 V	2.5 µA	693 mW m^−2^	[243]
Cellulose	Triboelectric layer	Ag	21.9 V	0.73 µA	7.68 µW cm^−2^	[244]
Cellulose paper	Triboelectric layer	Au	120 V	2.5 mA m^−2^	72.5 mW m^−2^	[245]
Cellulose nanopaper	Triboelectric layer	PEDOT:PSS	75 V	18 µA	≈84.4 µW	[108]
CNF	Triboelectric layer	ITO‐coated PET	101 V	9.2 µA	13.3 W m^−2^	[246]
CNF	Triboelectric layer	ITO‐coated PET	32.8 V	35 µA	0.56 mW	[107]
CNF	Triboelectric layer	ITO‐coated PET	60.6 V	7.7 µA	2.33 W m^−2^	[247]
Nitro‐CNF, methyl‐CNF	Triboelectric layer	Cu	8 V	9 µA	—	[109]
CCP, NCM	Triboelectric layer	Cu	196.8 V	31.5 µA	16.1 W m^−2^	[110]
Chitosan	Triboelectric layer	Al	13.5 V	42 nA	2.1 µW m^−2^	[112]
Starch	Triboelectric layer	Al	1.2 V	—	170 mW m^−2^	[111]
Sodium alginate	Triboelectric layer	Li, Al	1.47 V	3.9 nA	3.8 mW m^−2^	[113]
Calcium alginate	Triboelectric layer	Al	33 V	150 nA	9.5 µW	[44]
SF	Triboelectric layer	ITO‐coated PET	213.9 V	—	68.0 mW m^−2^	[248]
SF	Triboelectric layer	Graphite	666 V	174.6 µA	412 µW cm^−2^	[249]
SF	Triboelectric layer	Al	—	—	4.3 mW m^−2^	[114]
SF	Triboelectric layer	Ag nanowires	110 V	0.1 µA	2 mW cm^−2^	[250]
Gelatin	Triboelectric layer	Mg	500 V	—	5 W m^−2^	[87]
Leaf	Triboelectric layer	Cu	1000 V	60 µA	17.9 mW	[237]
Tea leaves	Triboelectric layer	Al	792 V	42.8 µA	488.88 µW cm^−2^	[251]
Rice paper	Triboelectric layer	Conductive ink	244 V	6 µA	37.64 µW cm^−2^	[252]
Lignin‐starch composite	Triboelectric layer	Cu	1.04 V cm^−2^	3.96 nA cm^−2^	173.5 nW cm^−2^	[253]
Cellulose, SF, egg white, chitin, rice paper	Triboelectric layer	Mg	55 V	0.6 µA	21.6 mW m^−2^	[115]

Abbreviations: CCP: crepe cellulose paper; NCM: nitrocellulose membrane; HPC: hydroxypropyl cellulose.

**Figure 4:**
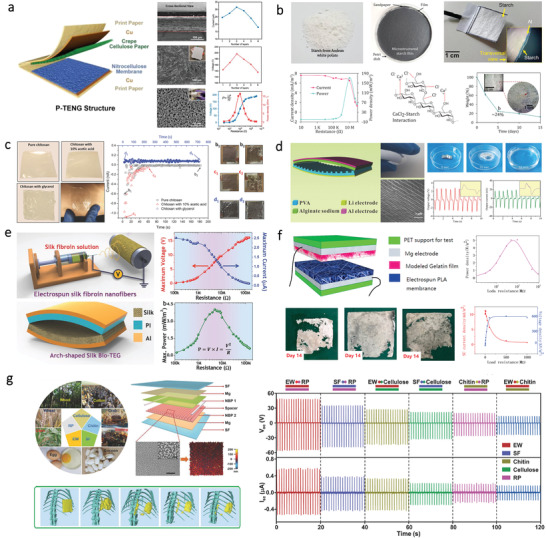
TENGs based on different kinds of bio‐origin materials. a) A paper‐based TENG based on CCP and NCM. Reproduced with permission.^[^ ^110^ ^]^ Copyright 2019, Elsevier. b) A bio‐TENG based on starch polymer electrolyte. Reproduced with permission.^[^ ^111^ ^]^ Copyright 2019, Elsevier. c) Biodegradable and flexible TENG based on chitosan biopolymers. Reproduced with permission.^[^ ^112^ ^]^ Copyright 2018, Wiley‐VCH. d) A recyclable and green TENG using SA film as one of the friction materials. Reproduced with permission.^[^ ^113^ ^]^ Copyright 2016, Wiley‐VCH. e) A bio‐TENG based on SF nanofiber film obtained by electrospinning. Reproduced with permission.^[^ ^114^ ^]^ Copyright 2016, Wiley‐VCH. f) A fully biodegradable TENG based on nanostructured gelatin films. Reproduced with permission.^[^ ^87^ ^]^ Copyright 2018, Elsevier. g) Fully bioabsorbable TENG based on five natural bio‐origin materials. The “triboelectric series” of these materials is ranked. Reproduced with permission.^[^ ^115^ ^]^ Copyright 2018, Wiley‐VCH.

For example, Yao et al. used a CNF film as one active layer and paired it with a fluorinated ethylene propylene (FEP) film to assemble a TENG.^[^ ^107^ ^]^ This CNF‐based TENG can generate an output voltage of about 30 V and output current around 90 µA. However, the FEP film is non‐biodegradable, which may cause environmental pollution. To address this, Gao et al. designed a fully‐degradable TENG based on CNF film and polylactic acid (PLA) film.^[^ ^108^ ^]^ Results show that the CNF and PLA film can be fully degraded in natural soil without causing any pollutants to the environment. Due to the chemical structure of cellulose, it is usually used as positive parts of the TENG, the negative parts of the TENG are always synthetic polymers. To prepare tribonegative materials using cellulose, Yao et al. proposed chemical modification methods by attaching nitro groups and methyl groups to cellulose molecules, aiming to tune the triboelectric polarity of the cellulose.^[^ ^109^ ^]^ The fabricated TENG using nitrocellulose film as tribopositive layer and methylcellulose film as tribonegative layer shows a voltage output of around 8 V and a current output of 9 µA. To further simplify the fabrication process and enhance the output performance, Chen et al. reported a TENG using crepe cellulose paper (CCP) combined with a nitrocellulose membrane (NCM),^[^ ^110^ ^]^ as shown in **Figure** **4**a. The opposite tribopolarities and different microstructure of CCP and NCM endows the TENG with outstanding triboelectric performance. Ccorahua et al. developed a TENG with microstructure surfaces using starch.^[^ ^111^ ^]^ As shown in Figure 4b, the starch solution obtained from Andean white potatoes was coated on the surfaces of sandpaper to obtain a microstructure surface. Results show that the TENG achieves a voltage output of 1.2 V, with a power density of 170 mW m^–2^. The biodegradation of the starch‐based film was also investigated. Results show that by diving the film in compost, it can be mostly degraded after 15 days. Although TENG based on starch shows good biodegradability, the output performance is quite low. As another kind of polysaccharide, chitosan also shows great potential in fabricating low‐cost and biodegradable TENG. Wang et al. developed a TENG using engineered and laser‐processed chitosan biopolymer.^[^ ^112^ ^]^ As shown in Figure 4c, different nanocomposite films based on chitosan are fabricated. Results show that the chitosan‐based nanocomposite processed with 10% acetic acid exhibits the highest output, with the voltage and current about 13.5 V and 42 nA, respectively. Furthermore, the degradation of these chitosan‐based nanocomposite films was also investigated. Results suggest that by engineering the physical and chemical features of chitosan, the biodegradability of the TENG can be controlled, which confirms their potential as sustainable power sources. Even though the TENG mentioned above shows degradability, the degradation process needs a long time, which may not meet the requirements for transient applications. As such, Zhang et al. developed a transient TENG based on PVA and sodium alginate (SA) that is fast‐soluble and recyclable,^[^ ^113^ ^]^ as shown in Figure 4d. This transient TENG using lithium (Li) and aluminum (Al) as a current collector can be fully dissolved by water. The entire device can be fully decomposed within 10 min after putting it into water.

Except for polysaccharides, protein‐based natural materials can also be applied for the fabrication of TENG. For instance, Kim and coworkers demonstrated a TENG based on SF in 2016.^[^ ^114^ ^]^ The SF film fabricated by an electrospinning method shows a nanofiber network structure, which is useful for enhancing the triboelectric power generation performance. As illustrated in Figure 4e, the TENG consists of SF nanofiber film and polyimide (PI) film, with Al foil as the current collector. This TENG could achieve an output power density of about 4.3 mW m^−2^. To further fabricate a fully‐degradable TENG, Luo and coworkers proposed a TENG using gelatin film and electrospun PLA film as the friction layer, magnesium (Mg) as a current collector, as shown in Figure 4f.^[^ ^87^ ^]^ To enhance the output performance, the gelatin film was fabricated by using a sandpaper template to create rough surfaces. The optimized output voltage and current density up to 500 V and 10.6 mA m^−2^ can be achieved. Also, the TENG can be fully degraded in water within 40 days. To reduce the usage of synthetic polymer, Jiang et al. developed a fully biodegradable TENG based on five different natural bio‐origin materials.^[^ ^115^ ^]^ As illustrated in Figure 4g, the “triboelectric series” of these five natural bio‐origin materials have been sequenced, which offers basic information for selecting materials of the TENG. Through different combinations of these natural materials, various outputs could be achieved. In addition, this TENG is fully degradable and resorbable in vivo, holding great promise as a power source for biomedical applications.

#### 3.1.2 Piezoelectric Nanogenerator

The concept of piezoelectric nanogenerator (PENG) was proposed by Wang et al. in 2006.^[^ ^116^ ^]^ Based on piezoelectric ZnO nanowires (NWs), they demonstrated that PENG could convert random mechanical energy such as tiny physical motions into electricity. The working mechanism of PENG can be ascribed to the piezoelectric effect ZnO NWs, which is associated with the generation of electric dipole moments in solids. When an external force is applied to the material, the negative and positive charge centers would separate, leading to the formation of a piezoelectric potential. By connecting an external load to the material, free electrons would flow via the external circuit to achieve a new equilibrium state.^[^ ^103^ ^]^ In this way, by applying a dynamic external force, a current pulse can be generated continuously. This piezoelectric potential generation mechanism of ZnO is also applicable to other piezoelectric materials.^[^ ^117^ ^]^ Generally, there are two key factors for the fabrication of PENG: materials selection and device structure design.^[^ ^118^ ^]^ So far, many attempts had been made to fabricate PENG by employing various materials such as inorganic nanomaterials like BaTiO_3_,^[^ ^119^ ^]^ ZnO,^[^ ^120^ ^]^ and KNbO_3_.^[^ ^121^ ^]^ Piezoelectric organic polymers like poly(vinylidene fluoride) (PVDF)^[^ ^122^ ^]^ and poly(vinylidene fluoride‐trifluoroethylene) (P(VDF‐TrFE))^[^ ^123^ ^]^ are also widely utilized for fabricating flexible PENG for wearable applications. Nevertheless, most polymer‐based piezoelectric material exhibits bad piezoelectric performance, which hinders their wide applications for the fabrication of high‐performance PENGs. As such, piezoelectric composites that comprise piezoelectric fillers and flexible matrix have been developed to fabricate high‐performance and flexible PENGs. Natural bio‐origin materials such as cellulose, silk, fish gelatin with piezoelectricity, flexibility, and biocompatibility hold great promise to act as a flexible matrix for the development of eco‐friendly PENG for multiple applications (**Table** **2**).

**Table 2:** Summary of PENG based on natural bio‐origin materials

Natural materials	Functions of the material	Output voltage	Output current	Power density	Piezoelectric coefficient	Ref.
Cellulose	Substrate	4.8 V	14.4 mA	1.3 mW mm^−2^	—	[254]
BC	Matrix with piezoelectric material	1.5 V	80 nA	60 nW cm^−2^	65.5 pm V^−1^	[125]
CNC	Piezoelectric material	60 V	—	—	—	[124]
CNC	Piezoelectric material	6.3 V	—	—	—	[255]
Chitin nanofiber	Piezoelectric material	22 V	0.12 µA	—	9.49 pC N^−1^	[256]
Chitin films	Piezoelectric material	1.04 V	—	184 nW cm^−2^	3.986 pm V^−1^	[257]
SF	Matrix with piezoelectric material	2.2 V	—	—	270 pC N^−1^	[126]
Spider silk fiber	Piezoelectric material	21.3 V	0.68 µA	4.56 µW cm^−2^	0.36 pm V^−1^	[258]
Collagen nanofibrils	Piezoelectric material	2 V	20 nA	0.75 mW m^−2^	−3 pC N^−1^	[127]
Eggshell membrane	Piezoelectric material	26.4 V	1.45 µA	11.9 µW cm^−2^	23.7 pC N^−1^	[259]
Fish scale	Piezoelectric material	4 V	1.5 µA	1.14 µW cm^−2^	−5 pC N^−1^	[260]
Prawn shell	Piezoelectric material	26.4 V	1.45 µA	11.9 µW cm^−2^	−2 pC N^−1^	[261]

**Figure 5:**
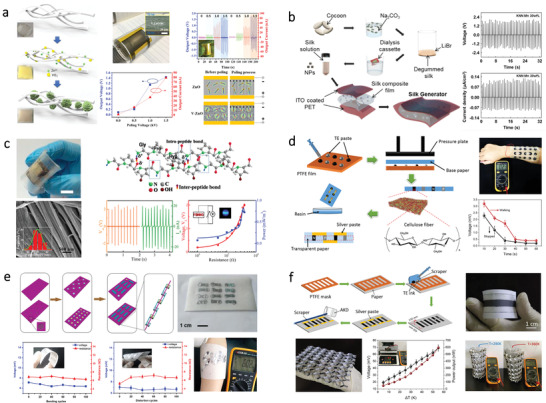
PENGs and TEGs based on different bio‐origin materials. a) Flexible PENG assembled from vanadium doped ZnO (V‐ZnO) mircoflowers in BC matrix. Reproduced with permission.^[^ ^125^ ^]^ Copyright 2018, Elsevier. b) SF‐based biodegradable composite‐type nanogenerators. Reproduced with permission.^[^ ^126^ ^]^ Copyright 2015, Elsevier. c) Fish skin as self‐poled natural piezoelectric material for constructing a nanogenerator. Reproduced with permission.^[^ ^127^ ^]^ Copyright 2019, American Chemical Society. d) Flexible TEG based on transparent cellulose paper for energy harvesting. Reproduced with permission.^[^ ^132^ ^]^ Copyright 2019, American Chemical Society. e) Silk fabric‐based wearable TEG for energy harvesting from the human body. Reproduced with permission.^[^ ^133^ ^]^ Copyright 2016, Elsevier. f) A TEG fabricated with a Bi_2_Te_3_/BC nanofiber. Reproduced with permission.^[^ ^134^ ^]^ Copyright 2019, Royal Society of Chemistry.

For example, Fu et al. developed a PENG using PVDF and CNC fibrous composites via an electrospinning method.^[^ ^124^ ^]^ Compared with pure PVDF fibers, PVDF/CNC composites possess higher content of *β*‐phase due to the addition of rod‐liked CNC, which improves the piezoelectric performance of the PENG. In addition to CNC, BC is also a kind of high crystallinity cellulosic material. Zhang and coworkers designed a hybrid cellulose paper via in situ assembly of vanadium‐doped ZnO (V‐ZnO) microflowers on a BC substrate.^[^ ^125^ ^]^ As shown in **Figure** **5**a, V‐ZnO grows randomly in the BC matrix during the growing process, which enhances the performance of the flexible PENG. Results show that an output voltage of 1.5 V and a current density of 80 nA cm^–2^ is obtained, respectively. In addition to cellulose, other bio‐origin materials such as SF can also be used as a biocompatible matrix for the fabrication of PENG. For instance, Kim et al. produced a flexible PENG by combining SF with ferroelectric nanoparticles.^[^ ^126^ ^]^ As illustrated in Figure 5b, mixed SF‐nanoparticles solution is drop‐casted onto a flexible ITO/PET substrate, the other ITO/PET film attached to the composite layer serves as the top electrode. This fabricated PENG exhibits an output voltage of 2.2 V and a current density of 0.12 mA cm^–2^. Moreover, due to the biodegradability of SF, the lifetime of the PENG is also controllable in water.

Except being used as a flexible matrix, some bio‐origin materials such as collagen nanofibrils also can be used as piezoelectric material separately without other synthetic materials. Gohsh et al. developed a flexible PENG using fish skin (Figure 5c).^[^ ^127^ ^]^ The piezoelectricity of collagen nanofibrils can be ascribed to the hydrogen bonding within the polypeptide chains. The self‐assembled collagen nanofibrils exhibit stable crystalline structures as well as nonlinear electrostriction effect without any electrical poling treatment. Results show that this PENG could generate open‐circuit voltage around 2 V, with short‐circuit current around 20 nA.

#### 3.1.3 Thermoelectric Generator

A thermoelectric generator (TEG) is a solid‐state device that converts thermal flux into electrical energy based on the principles of Seebeck effect. Typically, a thermoelectric module comprises both n‐ and p‐type semiconducting materials that connected electrically in series and thermally in parallel.^[^ ^128^ ^]^ If there exists a temperature gradient across the module, electricity could be generated.^[^ ^129^ ^]^ Owing to the temperature gradient, carriers would drift from the cold side to the hot side, leading to the formation of an electric field. The electrochemical potential can generate power and drive external loads.^[^ ^130^ ^]^ In recent years, TEG has attracted great interest due to its ability to generate power from renewable resources. Particularly, flexible TEG that can be attached to a human body for powering wearable electronics has been extensively studied.^[^ ^131^ ^]^ So far, great efforts have been made to improve the flexibility of TEG as well as enhancing its energy conversion efficiency. To date, most substrates used in flexible TEG are based on synthetic polymer materials such as polydimethylsiloxane (PDMS), polyethylene glycol terephthalate (PET), and PI. Although the TEG fabricated with these polymer materials exhibits flexibility, the poor biocompatibility of these materials limits their applications. As such, natural bio‐origin materials with great flexibility, biodegradability, and large availability are considered as ideal substrates for TEG (**Table** **3**).

**Table 3:** Summary of TEG based on natural bio‐origin materials

Natural materials	Function of materials	Temperature difference	Output voltage	Energy density	Ref.
Cellulose	Substrate	35 K	8.3 mV	10 nW	[132]
Cellulose	Scaffold	16 K	0.3 mV	—	[262]
Cellulose fiber	Substrate	15 K	2 mV	0.2 µW	[263]
CNF	Binder	50 K	8.4 mV	1.5 nW	[264]
CA	Polymer matrix	10 K	—	2.28 µW	[263]
CA	Polymer matrix	30 K	—	2.48 µW	[265]
BC	Binder	55 K	70.5 mV	596 nW	[134]
Silk fabric	Substrate	30 K	10 mV	15 nW	[133]

For example, Zhao et al. developed a flexible TEG on a cellulose paper substrate, as shown in Figure 5d.^[^ ^132^ ^]^ The paper‐based TEG exhibited great mechanical flexibility. The TEG retained an output voltage of about 8.3 mV at a temperature difference of 35 K after 1000 bending cycles. To further promote wearable applications of flexible TEG, Zhu et al. developed a wearable TEG using commercial silk fabrics for power generation by harvesting waste heat from the human body.^[^ ^133^ ^]^ As shown in Figure 5e, the TEG can convert thermal energy into electricity efficiently. The output voltage and power show little changes during 100 bending and twisting cycles. Besides, combined with traditional printing technology, the TEG can be designed into patterns on clothes, holding great promise in next‐generation wearable devices.

In addition to being used as the substrates, some natural bio‐origin materials can also act as a binder to enhance the performance of TEG. For instance, a honeycomb‐like TEG was designed using thermoelectric ink that was formulated by Bi_2_Te_3_ and BC.^[^ ^134^ ^]^ Herein, BC acts as the binder material, which could improve the electrical conductivity as well as the energy conversion efficiency of the TEG. At a temperature difference of 55 K, the TEG achieves an output voltage of 70.5 mV, with an output power of 596 nW.

#### 3.1.4 Solar Cell

The solar energy that provides basic energy for all living creatures is considered as the most fundamental renewable energy source on earth. A solar cell (SC) is a device that can convert solar radiation into electricity directly. The first generation of SC that is made of monocrystalline silicon needs high fabrication cost, which hinders its commercial availability. The thin film SC with thickness ranging from a few nanometers to tens of microns is referred as the second generation, which consists of thin films made of CdTe, CIGS, and amorphous silicon. Even though these thin‐film SCs are more flexible and light‐weight, the power conversion efficiency (PCE) is relatively low. As such, other thin‐film technologies are being researched currently, known as the third generation of SC, which mainly includes dye‐sensitized solar cell (DSSC), organic solar cell (OSC), and the perovskite solar cell (PSC). Although great progress has been made, core improvements are still needed to make SCs economically feasible. Particularly, developing SCs with flexibility and biodegradability are essential for environmentally‐friendly applications. At present, natural bio‐origin materials are considered as an emerging candidate for fabricating new generation SCs (**Table** **4**).

**Table 4:** Summary of solar cells based on natural bio‐origin materials

Natural materials	Function of materials	Solar cell type	Output mA cm^−2^	PCE	Ref.
Cellulose	Electrode	OSC	6.79	2.01%	[139]
CNF	Substrate	OSC	—	5.88%	[135]
CNF	Substrate	OSC	5.0	0.4%	[266]
CNF	Substrate	OSC	9.58	3.2%	[267]
CNF	Electrolyte	DSSC	15.2	8.25%	[268]
CNF	Electrolyte	DSSC	11.7	4.7%	[142]
CNC; CNF	Substrate	OSC	3.5; 2.0	1.4%; 0.5%	[269]
CNC	Substrate	OSC	7.3	4.0%	[137]
CNC	Substrate	OSC	7.5	2.7%	[136]
HPC	Electrolyte	DSSC	13.73	5.79%	[141]
Wood	Light management layer	OSC	20.17	13.49%	[270]
Wood	Light management layer	OSC	19.78	14.41%	[146]
Chitosan	Cathode interlayers	OSC	17.3	9.34%	[140]
Chitosan	Electrolyte	DSSC	2.71	0.78%	[143]
Rice starch	Electrolyte	DSSC	0.828	0.35%	[145]
Rice starch	Electrolyte	DSSC	2.40	0.78%	[144]
SF	Substrate	OSC	14.56	6.62%	[138]
SF	LDS	OSC	7.7	2.8%	[271]

Abbreviations: LDS: luminescent downshifting film.

**Figure 6:**
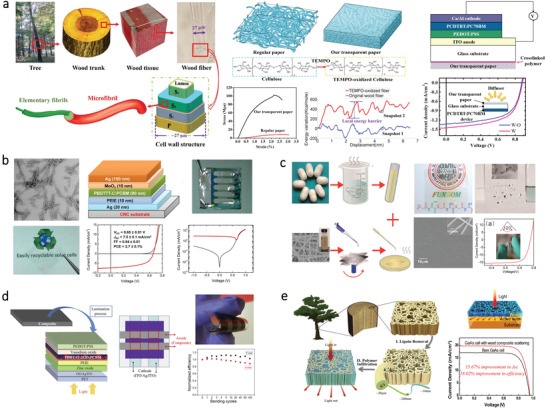
SCs based on different kinds of bio‐origin materials. a) Transparent CNF film as substrates for SC. Reproduced with permission.^[^ ^135^ ^]^ Copyright 2014, American Chemical Society. b) CNC film as the substrate for OSC. Reproduced with permission.^[^ ^136^ ^]^ Copyright 2013, Springer. c) SF as the substrate for SC. Reproduced with permission.^[^ ^138^ ^]^ Copyright 2014, American Chemical Society. d) Cellulose and sprayed graphene nanoplatelets as top electrode through lamination for OSC. Reproduced with permission.^[^ ^139^ ^]^ Copyright 2018, Elsevier. e) Transparent wood composite for light management in SC. Reproduced with permission.^[^ ^146^ ^]^ Copyright 2016, Elsevier.

The natural bio‐origin materials such as cellulose and SF have been successfully used as flexible substrates for SCs as evidenced by multiple publications. Particularly, a cellulose‐based substrate with high transparency was extensively utilized due to its ability to enhance the light scattering effect. For example, Fang et al. proposed a TEMPO‐oxidized CNF film made from wood fibers, which was used as a transparent substrate for the construction of OSC, as illustrated in **Figure** **6**a.^[^ ^135^ ^]^ They demonstrated that the high transparency and optical haze of TEMPO‐oxidized CNF film can be advantageously exploited to improve the PCE of an OSC by 10%.

Besides, the natural bio‐origin materials can be integrated with conductive materials to prepare flexible and conductive substrates. For instance, Zhou et al. produced a recyclable OSC on a transparent CNC substrate.^[^ ^136^ ^]^ As shown in Figure 6b, The device was fabricated by first depositing a semi‐transparent silver layer on the CNC film, which had nanometer‐level roughness that eliminated the need for planarization. The SCs exhibited excellent rectification in the dark and can achieve a PCE of 2.7%. Then, the same group reported another work that featured SCs with an enhanced PCE of 4.0%.^[^ ^137^ ^]^ The SCs were fabricated by depositing a conductive polymer on a CNC substrate via a film‐transfer lamination. Besides, Liu and co‐workers fabricated a flexible OSC by integrating SF with silver nanowires (AgNWs).^[^ ^138^ ^]^ As shown in Figure 6c, the substate consists of SF and AgNWs displayed a conductivity of ≈11 Ω sq^–1^ with transparency of over 80% at 600 nm. Moreover, compared with ITO on a synthetic plastic substrate, this substrate could retain its conductivity after being bent for 200 times due to the flexibility of SF.

In addition to be used as substrate materials, the natural bio‐origin materials can also serve as electrode components in SC. For example, Luca et al. developed a flexible cellulose‐graphene composite electrode as the top electrode of the SC via spray coating.^[^ ^139^ ^]^ As shown in Figure 6d, a scalable method was designed to deposit all layers of flexible polymers on the substrates. Results show that even after 100 bending cycles, the PCE of this SC remains stable. Zhang et al. employed chitosan as cathode interlayer materials in OSC.^[^ ^140^ ^]^ Results indicate that the PCE increased from 3.24% to 5.33% upon the addition of chitosan, suggesting the potentials of chitosan for working as a cathode interlayer material.

Besides, the natural bio‐origin materials are also widely used as electrolytes for DSSC. For instance, Khanmirzaei et al. prepared gel polymer electrolytes based on hydroxypropyl cellulose (HPC) for constructing DSSC.^[^ ^141^ ^]^ Miettunen et al. introduced a method by utilizing a porous nanocellulose aerogel for depositing electrolyte in DSSC.^[^ ^142^ ^]^ The nanocellulose aerogel acts as an absorbent for the electrolyte and can prevent the electrolyte from spreading on the substrate. Buraidah et al. prepared an electrolyte using chitosan blended with poly (ethylene oxide) (PEO) for constructing DSSC.^[^ ^143^ ^]^ Herein, chitosan is considered as a suitable host in the mixed electrolyte because it is rich in oxygen and nitrogen atoms. These atoms could act as electron donors and form dative bonding with cations, which gives chitosan the capability to host ionic conduction. Besides, rice starch also found its applications in polymer electrolytes for constructing DSSC. For instance, Khanmirzaei et al. prepared a polymer electrolyte by mixing rice starch with different iodide salts via a solution cast technique.^[^ ^144^ ^]^ Yogananda et al. reported a biopolymer gel electrolyte (GE) using rice starch and Li iodide salt with 100% water as solvent.^[^ ^145^ ^]^

In addition to the before‐mentioned functions, the natural bio‐origin materials can also be used for enhancing the light‐trapping ability of the inside active layer. For instance, Zhu et al. fabricated a transparent wood composite with high optical transmittance as well as a high haze in a broad wavelength, as shown in Figure 6e.^[^ ^146^ ^]^ This wood composite can serve as a broad range light management layer, which could enhance the overall PCE to 18%.

### 3.2 Energy Storage Device

#### 3.2.1 Supercapacitor

A supercapacitor, also known as an electrochemical capacitor or ultracapacitor, is extensively used in portable electronics and stationary energy storage. According to the storage mechanism, supercapacitor can be briefly classified as electrochemical double‐layer capacitors (EDLC), pseudocapacitor, and hybrid‐capacitor. EDLC is normally fabricated using nanoporous carbon material as the active electrode material. Pseudocapacitor is usually fabricated using electrodes based on conducting polymers or metal‐oxides, which combines both electrostatic and pseudocapacitive charge storage mechanisms. Structurally, a supercapacitor cell is composed of two electrodes with a separator between them. The separator that soaked in the electrolyte is used for preventing any electrical contact between the two electrodes, which should be ion‐permeable for ionic charge transfer. The electrolytes that providing ionic conductivity have different types including liquid electrolytes and solid/gel‐state electrolytes. Typically, the supercapacitor is required to be durable for long‐time usage. Whereas, for some specific cases, the supercapacitor is needed to be biodegradable after their service lifetime. Moreover, the fabrication of supercapacitors using biodegradable and nontoxic materials for implantable application is necessary. As such, natural bio‐origin materials hold great promise in the fabrication of supercapacitors, including electrodes, separator, and GE (**Table** **5**).

**Table 5:** Summary of supercapacitors based on natural bio‐origin materials

Materials	Function of the materials	Type	Areal capacitance	Energy density	Ref.
Cellulose	PANI/cellulose as electrodes	Asymmetric	160 F g^−1^	—	[272]
Cellulose	MWCNTs/PEDOT:PSS/cellulose as electrode	Symmetric	380 F g^−1^	13.2 Wh kg^−1^	[273]
CF	Substrate for electrode materials	Asymmetric	295 F g^−1^	—	[274]
CNF	Substrate for electrode materials	Asymmetric	100 mF cm^−2^	—	[148]
CNF	PANI/Ag/CNF aerogel as electrode	Symmetric	176 mF cm^−2^	—	[152]
CAF	Electrodes after carbonization	—	241.4 F g^−1^	—	[153]
BC	Electrodes after carbonization	Symmetric	224 F g^−1^	31.04 Wh kg^−1^	[154]
BC	Substrate for electrode materials	Symmetric	656 F g^−1^	—	[150]
BC	CNT/BC as electrode	Symmetric	50.5 F g^−1^	15.5 mWh g^−1^	[149]
BC	RGO/PEDOT:PSS/BC as electrode	Asymmetric	373 F g^−1^	—	[151]
Cellulose paper	Substrate for electrode materials	Symmetric	81 mF cm^−2^	15 μWh cm^−2^	[275]
Wood	Electrode and separator	Asymmetric	3600 mF cm^−2^	1.6 mW h cm^−2^	[161]
Wood, cellulose paper	Carbonized wood as anode; cellulose as separator	Asymmetric	3.723 F cm^−2^	0.69 mWh cm^−2^	[162]
Chitosan	GE	Symmetric	47 F g^−1^	—	[157]
Chitosan	GE	Asymmetric	10 mF cm^−2^	—	[156a]
Carboxylated chitosan	GE	Symmetric	45.9 F g^−1^	5.2 Wh kg^−1^	[159]
Chitosan and starch	GE	Symmetric	133 F g^−1^	—	[158]
Agarose	GE	Symmetric	1.6 mF cm^−2^	0.0083 μWh cm^−2^	[276]
Agarose	GE	Symmetric	286.9 F g^−1^	—	[277]

Abbreviations: CAF: cellulose acetate fibers.

**Figure 7:**
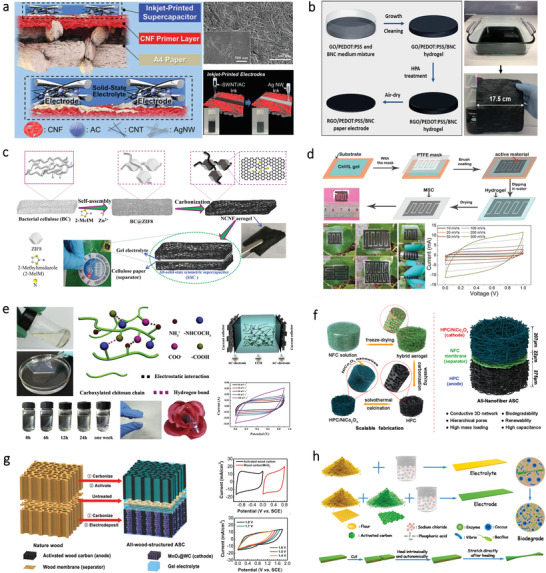
Supercapacitors based on different kinds of bio‐origin materials. a) CNF on cellulose paper as a substrate for flexible supercapacitors. Reproduced with permission.^[^ ^148^ ^]^ Copyright 2016, The Royal Society of Chemistry. b) BC as a layered matrix for electrodes of flexible supercapacitors. Reproduced with permission.^[^ ^151^ ^]^ Copyright 2017, The Royal Society of Chemistry. c) BC carbon aerogels as electrode materials for all‐solid‐state supercapacitor. Reproduced with permission.^[^ ^154^ ^]^ Copyright 2019, Elsevier. d) KOH‐saturated mesoporous cellulose membrane as polymer electrolyte for solid‐state supercapacitors. Reproduced with permission.^[^ ^155^ ^]^ Copyright 2017, Wiley‐VCH. e) Carboxylated chitosan hydrogel film for all‐solid‐sate supercapacitors. Reproduced with permission.^[^ ^159^ ^]^ Copyright 2019, Elsevier. f) All‐nanofiber asymmetric supercapacitor assembled using nanocellulose‐derived materials. Reproduced with permission.^[^ ^160^ ^]^ Copyright 2019, American Chemical Society. g) An all‐wood‐structured asymmetric supercapacitor. Reproduced with permission.^[^ ^161^ ^]^ Copyright 2017, The Royal Society of Chemistry. h) Self‐healable flour as the main material of both electrolytes and electrodes for supercapacitor. Reproduced with permission.^[^ ^163^ ^]^ Copyright 2019, Elsevier.

Recently, cellulose has attracted great attention for fabricating flexible supercapacitors due to its ability to integrated with conductive materials. For example, Zheng et al. designed a paper‐based electrode for supercapacitors by directing drawing graphite on the cellulose paper. This paper‐based supercapacitor shows a capacitance of 2.3 mF cm^–2^.^[^ ^147^ ^]^ To better simplify the fabrication process and pattern the electrodes in a controlled manner, Choi et al. developed a solid‐state flexible supercapacitor using inkjet printing technology.^[^ ^148^ ^]^ The schematic illustration showing the architecture of the supercapacitor is presented in **Figure** **7**a. It is notable that CNF primer layers on top of A4 paper display nanoporous structures and identical surface roughness, thereby improving the electrical conductivity of the electrodes. Another type of cellulose, BC that consists of ribbon‐shaped ultrafine nanofibers, can also be used for supercapacitor electrodes due to its adequate porosity, strong tensile strength, and excellent water retention ability. For example, Kang and coworkers fabricated a flexible electrode by depositing CNT layers onto the BC film via a vacuum filtration process.^[^ ^149^ ^]^ Similarly, Li et al. proposed a supercapacitor based on flexible electrodes by depositing multiwalled carbon nanotubes (MWCNTs) and polyaniline (PANI) on the BC film.^[^ ^150^ ^]^ However, this work involves vacuum filtration methods, which is difficult for large‐scale production. To address this, Jiang et al. introduced a strategy by in‐situ incorporations of reduced graphene oxide (rGO) flakes and PEDOT:PSS into a BC matrix during its growing process for large‐scale fabrication of flexible BC‐based electrodes,^[^ ^151^ ^]^ as illustrated in Figure 7b. Here, BC served as a layered matrix for incorporation of GO. The obtained rGO/PEDOT:PSS/BC electrodes can be assembled into flexible supercapacitors, which displays remarkable energy storage performance as well as robustness. To further improve the performances of the supercapacitors, cellulose can be fabricated into 3D porous aerogels. For instance, Zhang et al. developed a flexible aerogel supercapacitor using CNF and conductive nanoparticles.^[^ ^152^ ^]^ The CNF‐based aerogel exhibits high porosity. Besides, cellulose with abundant hydroxyl moieties can be transformed into heteroatom‐doped carbon materials, thereby allowing diverse functionalization to design new electrodes. For example, a nitrogen‐doped carbon aerogel is fabricated through carbonizing polypyrrole‐coated CNF,^[^ ^153^ ^]^ which can serve as the electrodes for supercapacitor. Furthermore, Chen et al. developed a scalable approach for the fabrication of highly porous aerogels by in situ growth of zeolitic imidazolate frameworks (ZIF8) nanocrystals on BC, as shown in Figure 7c.^[^ ^154^ ^]^ Due to the distinctive porous structures of the aerogel, the flexible supercapacitor displays a capacitance of 224 F g^−1^ at 0.5 A g^−1^ as well as long‐term durability.

Apart from serving as electrode materials, the bio‐origin materials can be directly fabricated into GE as well. Zhao et al. fabricated a flexible and transparent mesoporous cellulose film (mCel‐film) by phase‐inversion technology using ionic liquid (IL) as the solvent, as shown in Figure 7d.^[^ ^155^ ^]^ The obtained flexible mCel‐film with porous structures possesses diverse properties such as electrolyte uptake, ionic conductivity, and thermal stability. By combining the mCel‐film with carbon electrodes, a solid‐state EDLC can be obtained, which shows a capacitance of 110 F g^−1^ at 1.0 A g^−1^. In addition to cellulose, the chitosan‐based hydrogel can also be used as electrolyte materials for supercapacitor.^[^ ^156^ ^]^ However, pure chitosan films often possess poor ionic and proton conductivity without modification. Therefore, different methods have been proposed to enhance the ionic conductivity of chitosan film by combining it with Li salts, plasticizers, and polymers. For instance, Sudhakar and coworkers prepared a biodegradable polymer electrolyte by mixing chitosan and PEG as the host polymer and lithium perchlorate (LiClO_4_) as a dopant.^[^ ^157^ ^]^ Based on this electrolyte, a supercapacitor with a specific capacitance of 47 Fg^–1^ can be obtained. In addition to PEG, they also fabricated biodegradable polymer electrolytes for supercapacitors by mixing starch with LiClO_4_ doped chitosan.^[^ ^158^ ^]^ Apart from adding additives, surface modification of chitosan can also enhance its properties. For instance, Yang et al. fabricated a GE with flexibility and transparency based on carboxylated chitosan hydrogel.^[^ ^159^ ^]^ As shown in Figure 7e, the obtained hydrogel film can be fabricated into an all‐solid‐state supercapacitor using carbon cloth as the current collector and activated carbon film as electrodes. This all‐solid‐state supercapacitor displays a specific capacitance of 45.9 F g^–1^ at 0.5 A g^–1^.

Flexible supercapacitors can also be fabricated by using bio‐origin materials as both electrodes and separators, holding great promise for fully biodegradable energy devices. Zhang et al. proposed a method for constructing supercapacitor with nanocellulose acting as both electrodes and separators (Figure 7f).^[^ ^160^ ^]^ The supercapacitor not only exhibits a high specific capacitance about 64.83 F g^−1^ at 0.25 A g^−1^, but also holds great promise for developing renewable energy‐storage devices. Additionally, wood‐derived materials have attracted great attention recently due to the straight channels, which allows fast mass transport along with the channel directions. Recently, Chen and coworkers reported a wood‐based asymmetric supercapacitor (Figure 7g).^[^ ^161^ ^]^ The anode, separator, and cathode are all made from natural wood, which possesses intrinsic anisotropic structures with multitudinous open channels along the growth direction. Owing to the high mass loading of the electrodes, this asymmetric supercapacitor shows a high areal capacitance of 3.6 F cm^–2^ at 1 mA cm^–2^ and an energy density of 1.6 mW h cm^–2^. Similarly, Wang et al. reported a sustainable strategy for fabricating an all‐solid‐state supercapacitor based on wood and cellulose.^[^ ^162^ ^]^ The supercapacitor displays an areal capacitance of 3.723 F cm^–2^ at a current density of 1.0 mA cm^–2^. To further endow supercapacitor with stretchability and self‐healing capability, Hu et al. fabricated a self‐healable supercapacitor by utilizing flour as the main material, as shown in Figure 7h.^[^ ^163^ ^]^ The obtained self‐healable and supercapacitor stretchable can be degradable after full utilization. These supercapacitors fabricated with bio‐origin materials open new opportunities for next‐generation flexible energy storage devices.

#### 3.2.2 Battery

A battery is composed of three functional components: two electrodes, an electrolyte, and a separator. It is another kind of energy device with high operation voltage and energy density. Bio‐origin materials are widely used as components of battery including electrodes (e.g., active materials, binders, and structural support), electrolytes, and separators (**Table** **6**).

**Table 6:** Summary of battery based on natural bio‐origin materials

Materials	Function of the materials	Type	Discharge capacity	Energy density	Ref.
CNF	Separator	Li‐ion	—	—	[165]
CNF	Separator	Li‐ion	138 mAh g^−1^	—	[164]
CNF	Separator	Li‐sulfur	947 mAh g^−1^	—	[278]
CNF	Electrode	Li‐LFP	8.8 mAh cm^−2^	538 Wh L^−1^	[173]
Cellulose	GE	Li‐ion	145 mAh g^−1^	—	[170]
Methyl cellulose	GE	Li‐ion	150 mAh g^−1^	—	[279]
Methyl cellulose	GE	Li‐ion	140 mAh g^−1^	—	[280]
BC	Solid electrolyte	Li metal	178.4 mAh g^−1^	—	[167]
Lignocellulose	GE	Li‐sulfur	1167.7 mAh g^−1^	—	[169]
Chitosan	GE	Li‐ion	146.8 mAh g^−1^	—	[281]
Wood	Electrode	Li‐ion	7.6 mAh cm^−2^	26 mWh cm^−2^	[174]
Wheat flour	Solid electrolyte	Li‐ion	132.3 mAh g^−1^	—	[282]
Alginate	GE	Mg‐MoO_3_	6.5 mAh cm^−2^	—	[220]
Soy protein	GE	Li‐ion	118.2 mAh g^−1^	—	[171]
SF	Separator	Li‐ion	126 mAh g^−1^	—	[166]
SF	SF‐PPy as electrode	Mg–air battery	3.79 mAh cm^−2^	4.70 mW h cm^−2^	[283]

A separator is a component placed between two battery electrodes to serve as a medium for ions transfer, which is a vital component of a battery. It is expected to possess excellent mechanical and thermal stability as well as ionic conductivity. Typically, the separator is composed of a polymer membrane soaking in the electrolyte solution. The commonly used separators are usually synthetic polymers such as polypropylene (PP), polyethylene oxide (PEO), PVDF, etc. Whereas, extensive usage of these synthetic polymers may cause negative environmental impact, which makes it highly necessary to replace them with natural polymers while maintaining the battery performance at the same time. Therefore, various methods have been developed recently to prepare separators using natural bio‐origin materials such as cellulose, lignin, and SF. For instance, Chun et al. fabricated a CNF paper as the separator membrane for Li‐ion batteries.^[^ ^164^ ^]^ The CNF with a nanometer‐scale diameter and micrometer‐scale length exhibits excellent mechanical properties, which endows the paper with a nanoporous structure.

**Figure 8:**
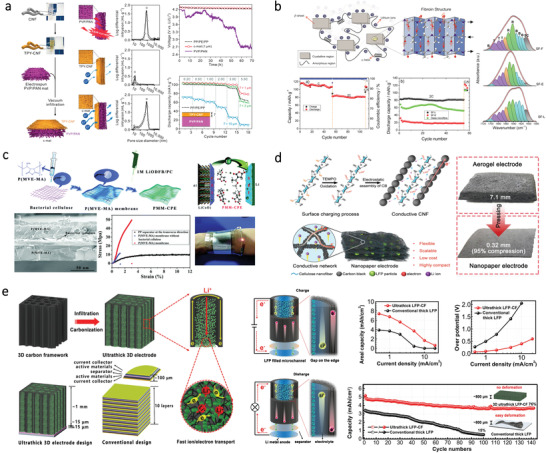
Batteries based on different kinds of bio‐origin material. a) CNF film as battery separators. Reproduced with permission.^[^ ^165^ ^]^ Copyright 2016, American Chemical Society. b) SF as separators for Li‐ion batteries. Reproduced with permission.^[^ ^166^ ^]^ Copyright 2018, American Chemical Society. c) Ultra‐strong BC as supporting material for polymer electrolytes for Li metal battery. Reproduced with permission.^[^ ^167^ ^]^ Copyright 2018, The Royal Society of Chemistry. d) Conductive cellulose nanofiber enabled thick electrode for Li‐LFP batteries. Reproduced with permission.^[^ ^173^ ^]^ Copyright 2018, Wiley‐VCH. e) Carbonized natural wood as an ultrathick 3D current collector for Li‐ion batteries. Reproduced with permission.^[^ ^174^ ^]^ Copyright 2017, Wiley‐VCH.

Kim et al. developed porous membranes based on CNF with synergistically coupled chemical activity.^[^ ^165^ ^]^ As shown in **Figure** **8**a, the separator comprises two layers, where a terpyridine (TPY)‐functionalized CNF nanoporous thin film covers a thick microporous film obtained by electrospinning of polyvinylpyrrolidone (PVP) and polyacrylonitrile (PAN). The unique nanoporous structure of TPY‐CNF could balance the trade‐off between leakage current and ion transport rate, resulting in higher discharge rate capability. Besides, Pereira et al. developed membranes based on SF with different surface morphology.^[^ ^166^ ^]^ As illustrated in Figure 8b, due to the efficient interaction between Li^+^ and the dipole moments of the antiparallel *β*‐sheet structure of SF, the ion movement mobility was greatly enhanced.

The batteries using the separator membrane mentioned above are based on liquid electrolytes. In contrast to liquid electrolytes, gel‐state and solid‐state electrolytes provide better security for fabricating portable battery. To this end, the natural bio‐origin materials that are biodegradable and renewable, have also been considered as promising building blocks for fabricating batteries based on gel‐ and solid‐state electrolytes. For instance, Dong et al. fabricated a multifunctional polymer electrolyte supported by BC for constructing a Li metal battery.^[^ ^167^ ^]^ As shown in Figure 8c, the obtained film shows a sandwich structure, where the ultra‐strong BC framework was sandwiched between two layers of poly (methyl vinyl ether‐alt‐maleic anhydride). Herein, BC acts as the supporting material for enhancing the mechanical strength of the polymer electrolyte membrane. Similarly, Zhang et al. prepared a polymer membrane using HEC and PVDF as the matrix of GE.^[^ ^168^ ^]^ This membrane possessed high ionic conductivity as well as a high Li‐ion transfer rate at room temperature. However, the obtained composite membrane is not degradable due to the existence of PVDF. Then, Song et al. fabricated a GE using lignocellulose (LC).^[^ ^169^ ^]^ This biodegradable LC‐based GE is prepared with a simple process, which endows the corresponding batteries with application possibility. To further enhance the ionic conductivity and Li‐ion transference of the GE, Du et al. prepared a cellulose‐based gel membrane with high mechanical robustness via solution casting and one‐step crosslinking technique.^[^ ^170^ ^]^ The obtained membrane exhibits excellent mechanical strength, high ionic conductivity, and a large Li‐ion transference amount. Besides, electrolyte membranes can also be prepared by electrospinning method to obtain nanofiber structures. Zhu et al. fabricated a biodegradable composite membrane based on soy protein isolate (SPI) and PVA by electrospinning, which can be used as skeleton materials in GE of Li‐ion batteries.^[^ ^171^ ^]^ The GE displays high ionic conductivity, excellent durability, and good compatibility with the lithium electrode.

Besides, the natural bio‐origin materials can act as scaffolds for supporting electrode materials. By certain treatments, the porosity and specific surface area of the bio‐origin material can be improved significantly, which is advantageous for enhancing the performance and cycling stability of the battery. For example, Wang and coworkers prepared BC‐based carbon materials via a KOH‐activated pyrolysis process.^[^ ^172^ ^]^ Serving as the anode material for Li‐ion battery, the BC‐based carbon electrode exhibit a specific capacity of 857 mAh g^−1^ after 100 cycles at 100 mAh g^−1^. Besides, a thick electrode that is fabricated with electroactive materials also shows great potential in enhancing the energy density of batteries. Kuang et al. designed high‐loading thick electrodes based on a conductive nanofiber network, where negatively charged CNF are covered by neutral carbon black nanoparticles.^[^ ^173^ ^]^ As illustrated in Figure 8d, The obtained electrodes show a layer‐by‐layer structure, which exhibits mechanical flexibility as well as excellent electrical conductivity due to decoupled electron‐ion transfer routes. To further simplify the fabrication process for thick electrodes, Chen and coworkers proposed a lightweight carbon framework (CF) 3D current collector via direct carbonization of the natural wood slice.^[^ ^174^ ^]^ As shown in Figure 8e, the channels of the CF was first filled with commercial lithium iron phosphate (LFP) nanoparticles and epoxy, then it is carbonized to obtain a 3D electrode. Due to the structure of the multichannel CF, the ultrathick 3D electrode exhibits long‐life cycling and low deformability with improved mechanical properties, which provides opportunities for designing thick electrodes for high‐performance batteries.

### 3.3 Sensor

#### 3.3.1 Pressure Sensor

A pressure sensor that can generate signals under certain pressure is considered as an attractive candidate for boosting the development of sensing technology in modern society.^[^ ^175^ ^]^ Based on the different signal transduction mechanisms, pressure sensors can be divided into various types including resistive‐type, capacitive‐type, and piezoelectricity‐type.^[^ ^176^ ^]^ In addition, with the advancement of wearable devices, flexible pressure sensors have gained much attention due to their extensive applications in human‐machine interfaces, artificial intelligence, and e‐skin.^[^ ^177^ ^]^ In general, a flexible pressure sensor is usually fabricated by integrating conductive sensing materials with flexible substrates. To meet the demands for various applications, developing new materials and device fabrication methods are highly desirable to manipulate the mechanical and electrical properties for enhancing device performance.^[^ ^178^ ^]^ Polymers such as PET, PDMS, etc. have been widely used as flexible substrates. Nanomaterials including carbon nanotubes (CNTs), graphene, and metal nanowires are commonly utilized as conductive sensing materials.^[^ ^179^ ^]^ However, the non‐biodegradability of PET or PDMS may cause an environmental problem in the long run. Hence, it is highly desirable to develop approaches for the fabrication of pressure sensors with excellent sensitivity, flexibility as well as biocompatibility. As such, natural bio‐origin materials such as cellulose, SF etc. hold great promise for fabricating next generation environmentally‐friendly pressure sensors (**Table** **7**).

**Table 7:** Summary of sensors based on natural bio‐origin materials

Sensor type	Materials	Function of the material	Transduce mechanism	Linear range	Sensitivity	Ref.
Pressure sensor	Cellulose	3D scaffold	Resistance	0–5 kPa	58.9 kPa^−1^	[180]
	CNF	Components for hydrogel	Resistance	0–4 kPa	0.75 kPa^−1^	[182]
	CNF	Components for 3D sponge	Resistance	—	—	[262]
	CNF	Components for aerogel	Resistance	—	—	[284]
	Chitosan	Matrix material	Piezoelectricity	5–60 kPa	2.82 kPa^−1^	[285]
	SF	Sensing material after carbonization	Resistance	0.1–5.5 kPa	34.47 kPa^−1^	[183]
Strain sensor	CMC	Components for aerogel	Resistance	0–20%; 20–45%; 45–70%	0.65; 1.11, 1.58	[190]
	CNC	Tailoring the network of nanocomposite	Resistance	—	43.5	[286]
	CNC	Substrate	Resistance	0–0.8%	52.44	[188]
	CNs	Scaffold for nanocomposite	Resistance	—	4427	[287]
	BC	Components for aerogel	Resistance	—	21	[288]
	CP	Sensing material after carbonization	Resistance	0–1.35%; 1.35–4.85%	10.10; 3.69	[194]
	Silk fiber	The core of the fiber‐based sensor	Resistance	0–15%	14.5	[192]
Humidity sensor	Cellulose	CKF as the sensing material	Resistance	11.3–97.3% RH	—	[199]
	CNF	Sensing material	Microwave frequencies	55–100%RH	2.82 MHz/%RH	[289]
	CP	Substrate	Conductance	0–75% RH	—	[196]
	CMC	Sensing material	Photonic	5–40% RH	—	[290]
	CNC	CNC/GO as the sensing material	Capacitance	—	—	[198]
	SF	Sensing material	Photonic	—	—	[291]
	Collagen nanofibrils	Sensing material	Piezoelectric	50–90% RH	0.1287 µA/% RH	[292]

Abbreviations: CMC: carboxymethyl cellulose; CNs: cellulose nanowhisker; CKF: cellulose/KOH composite ionic.

**Figure 9:**
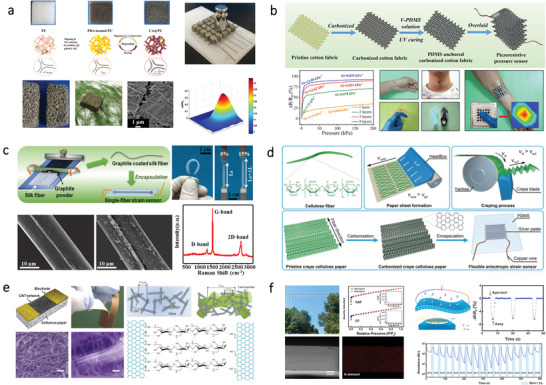
Sensors based on different kinds of bio‐origin materials. a) Piezoresistive pressure sensor based on CNF/AgNWs‐coated PU sponge. Reproduced with permission.^[^ ^181^ ^]^ Copyright 2019, American Chemical Society. b) Carbonized cotton fabric as a sensing material for multilayer piezoresistive pressure sensors. Reproduced with permission.^[^ ^184^ ^]^ Copyright 2019, Springer. c) Silk fiber as the core for supporting conductive materials for single fiber‐based strain sensors. Reproduced with permission.^[^ ^192^ ^]^ Copyright 2016, American Chemical Society. d) Carbonized crepe paper with aligned cellulose fibers as a sensing material for flexible strain sensor. Reproduced with permission.^[^ ^194^ ^]^ Copyright 2018, Wiley‐VCH. e) Cellulose paper as flexible substrates for a humidity sensor. Reproduced with permission.^[^ ^196^ ^]^ Copyright 2012, American Chemical Society. f) Flexible and transparent cellulose‐based ionic film for humidity sensing. Reproduced with permission.^[^ ^199^ ^]^ Copyright 2020, American Chemical Society.

For example, Luo et al. fabricated a flexible pressure sensor by combining the elastic cellulose sponge with conductive polypyrrole (PPy).^[^ ^180^ ^]^ The cellulose sponge was obtained by freeze‐drying of cellulose/NaOH/urea solution, followed by in‐situ chemical oxidative polymerization of PPy on its surface. This pressure sensor exhibited high conductivity, excellent sensitivity, and good durability. To further simplify the fabrication process, Zhang and coworkers fabricated conductive CNF/AgNWs (CA)‐coated polyurethane (PU) sponge based on a dip‐coating method (**Figure** **9**a).^[^ ^181^ ^]^ Due to the high modulus of cellulose, the conductive CA layer would easily generate a cracked structure under external compression, resulting in high sensing sensitivity. Besides, the addition of CNF not only enhances the dispersive ability of AgNWs but also the adhesion between conductive layer and PU skeleton. In addition to sponge structure, pressure sensors in hydrogel form that are stretchable and self‐healable can also be fabricated based on CNF. For instance, Jing and coworkers fabricated a self‐healing pressure sensor base on PVA/CNF hydrogel with a dual‐crosslinked network.^[^ ^182^ ^]^ Compared to PVA hydrogel, the PVA/CNF hydrogel with dynamic borate bonds and metal‐carboxylate coordination bonds exhibits excellent stretchability, self‐healing ability, and superior dimensional stability.

In the above researches, the natural bio‐origin material is used as an additive or skeleton as the support material for pressure sensors due to its poor conductivity. Recently, it has been reported that natural materials such as SF can be transferred into conductive graphitic structures via heating treatment. In contrast to synthetic conductive polymers, the obtained carbon‐based materials derived from natural materials have gained great attention due to their abundant resources, massive production ability, and environmental benignity. For example, Wang and coworkers fabricated a skin‐like pressure sensor with N‐doped carbon nanofiber network structures by carbonizing electrospun SF.^[^ ^183^ ^]^ The N‐doped carbon nanofiber network structure of the carbonized SF membrane endows the pressure sensor with high sensitivity (34.47 kPa^−1^), an ultralow detection limit (0.8 Pa), and a rapid response time about 16.7 ms. In addition, Zhang et al. reported a strategy to fabricate a multilayer piezoresistive pressure sensor using carbonized cotton fabric.^[^ ^184^ ^]^ As shown in Figure 9b, the carbonized cotton fabric with a multilayer structure could form 3D conductive network. By integrating with PDMS, the pressure sensor achieved a wide pressure detection range as well as high sensitivity.

#### 3.3.2 Strain Sensor

A strain sensor is a device that can transduce mechanical deformations into different electrical signals.^[^ ^185^ ^]^ Traditional strain sensors are usually fabricated using semiconductors or metal foils, which fail to meet the demands of wearable devices due to their rigidity as well as low sensing range.^[^ ^186^ ^]^ Therefore, great efforts have been made to fabricate flexible strain sensors by integrating nanomaterials (sensing elements) with elastic polymers (supporting materials).^[^ ^187^ ^]^ Besides, to eliminate the environmental problem caused by traditional elastic polymers, natural materials are recently used as building blocks for flexible strain sensors due to their biodegradability and compatibility.

Natural bio‐origin materials such as cellulose or silk can be used as flexible substrates or supporting scaffolds for constructing strain sensors. Meng et al. fabricated a strain sensor based on a flexible and water‐soluble CNC film.^[^ ^188^ ^]^ To further enhance the sensitivity, Xu and coworkers designed a flexible microstructured substrate using acetylcellulose.^[^ ^189^ ^]^ The porous acetylcellulose film can be easily fabricated by replicating a silica opal template, with rGO film serves as the strain‐sensing layer. In addition, cellulose can also be fabricated into aerogels for strain sensing by combing with conductive materials. For example, Huang et al. fabricated a compressive strain sensor based on graphene and CMC composite aerogel.^[^ ^190^ ^]^ Yao et al. reported a flexible strain sensor based on a Ag/CNF aerogel, which shows a GF of 1 up to 20% strain.^[^ ^191^ ^]^ In addition to aerogel, fiber‐based strain sensors can also be constructed using natural materials. For instance, Zhang et al. developed a fiber‐shaped strain sensor with a sheath‐core structure. As shown in Figure 9c, the strain sensor with exhibits a gauge factor of 14.5 within a strain range up to 15%.^[^ ^192^ ^]^

Recently, researchers have demonstrated that by carbonization process, the natural materials can be converted into carbonized conductive materials, which can be used as sensing materials of the strain sensor. For instance, Zhuo and coworkers fabricated a sensor for compression strain and pressure sensing by the carbonization of CNC/GO.^[^ ^193^ ^]^ Besides, a flexible strain sensor was fabricated using crepe paper made of aligned cellulose fibers by Chen and coworkers, as shown in Figure 9d.^[^ ^194^ ^]^ The fabricated strain sensor with a conductive network obtained by carbonization of crepe paper exhibits high flexibility, fast response time, and high durability.

#### 3.3.3 Humidity Sensor

A humidity sensor is a device for humidity measurement, which plays a key part in various applications.^[^ ^195^ ^]^ A high‐performance humidity sensor is expected to possess a wide detection range, rapid response time, and environmental stability. Materials such as porous ceramic and polymers that are sensitive to water molecules can be utilized as sensing materials for humidity sensors. Recently, some water‐sensitive natural bio‐origin materials are also considered as promising candidates for fabricating biocompatible and environmentally‐friendly humidity sensors.

Han and coworkers fabricated a humidity sensor by coating carboxylic acid‐functionalized single‐walled carbon nanotubes (SWCNTs) on cellulose paper, as shown in Figure 9e.^[^ ^196^ ^]^ Benefitting from the charge transport on a paper substrate, the obtained flexible humidity sensor shows higher sensitivity than that fabricated on a glass substrate. Also, cellulose‐based materials can be used as humidity sensing materials by combining with other materials. For instance, Shukla fabricated a moisture‐sensitive conductive film by grafting cellulose with PPy or PANI.^[^ ^197^ ^]^ Compared with single cellulose or PPy film, the grafting strategy endows the film with enhanced electrical properties and hygroscopic properties. In addition, CNC/GO composite can also act as a sensing material for the construction of a flexible humidity sensor by Kafy and coworkers.^[^ ^198^ ^]^ To further develop transparent and flexible humidity sensors, Wang et al. proposed a transparent humidity sensor based on a cellulose/KOH composite ionic film, as shown in Figure 9f.^[^ ^199^ ^]^ The obtained transparent and flexibility sensor displays high sensitivity, negligible hysteresis, and fast response time to humidity. This work provides a cost‐effective, easily processable, and environmentally‐friendly strategy for constructing sustainable and green humidity sensors.

### 3.4 Wireless Communication Device

#### 3.4.1 Radio‐Frequency Identification Device

**Figure 10:**
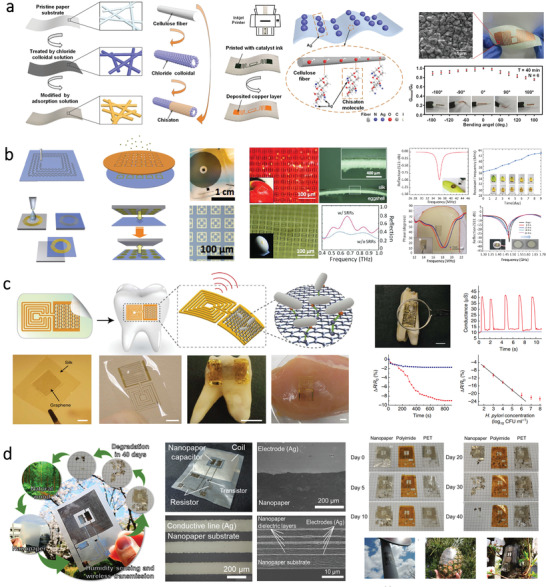
Wireless communication devices based on different kinds of bio‐origin materials. a) The fabrication process of flexible RFID devices on cellulose paper. Reproduced with permission.^[^ ^202^ ^]^ Copyright 2019, Wiley‐VCH. b) Wireless passive antennas on silk substrates for monitoring fruit ripening. Reproduced with permission.^[^ ^203^ ^]^ Copyright 2011, Wiley‐VCH. c) An inductively coupled radio frequency reader device on silk substrate for wireless monitoring of bacteria. Reproduced with permission.^[^ ^204^ ^]^ Copyright 2019, Springer Nature. d) A wireless transmission circuit based on transparent cellulose nanopaper. Reproduced with permission.^[^ ^210^ ^]^ Copyright 2020, American Chemical Society.

Radio‐frequency identification device (RFID) plays an important part in enabling identification technology in Internet‐of‐things (IoT).^[^ ^200^ ^]^ Typically, RFID tags are made of three pieces: a microchip that stores and processes information and modulates, an antenna for signal receiving/transmitting, and a supporting substrate. Particularly, for the application in a wearable device, the RFID is required to be flexible. Up till now, various flexible substrates such as PET, polyethylene naphthalate, and PI have been applied for fabricating RFID tags. In contrast to these synthetic substrates, cellulose paper that is widely available, inexpensive, and biodegradable has gained great attention for constructing disposable RFID devices.^[^ ^201^ ^]^ Cellulose paper serves as an insulating substrate would endow RFID tags with the following properties: 1) No need for extra coarsening process due to enough surface roughness. 2) Little absorption of electromagnetic energy owing to the low dielectric constant and loss tangent angle. As shown in **Figure** **10**a, Wang et al. fabricated an adhesive, low resistivity, and flexible metal antenna with RFID tags on paper substrates via a solution‐processing strategy.^[^ ^202^ ^]^ To enhance the adhesion between substrate and catalytic seed, the cellulose paper is immersed in a chitosan solution. The cellulose paper substrate not only ensures the flexibility of the RFID but also prevents ink infiltration loss. Results demonstrate that the fabricated flexible device displays excellent structural stability and relative conductivity after the bending test.

In addition to cellulose paper, silk is also widely used for the fabrication of flexible substrates. Tao and coworkers proposed a method for fabricating wireless passive antennas on a silk substrate.^[^ ^203^ ^]^ As illustrated in Figure 10b, a proof‐of‐concept application is demonstrated by monitoring fruit ripening using a flexible RFID‐like silk sensor. Overall, the results demonstrate that the RFID devices based on natural bio‐origin materials are promising green harmonic tags for practical applications. Similarly, using silk as a substrate, Mannoor et al. fabricated a wireless bacteria detection device by printing graphene on the water‐soluble silk substrate (Figure 10c).^[^ ^204^ ^]^ By self‐assembly of antimicrobial peptides onto graphene, detecting bacteria at single‐cell levels can be realized.

#### 3.4.2 Near‐Field Communication

Near‐field communication (NFC) is a wireless communication technology that allows two devices within a few centimeters to communicate.^[^ ^205^ ^]^ NFC technology is developed based on RFID, which allows a passive electronic tag to communicate with compatible hardware by inductive coupling between transmitting/receiving devices.^[^ ^206^ ^]^ The acting two parts of NFC can be sorted as initiators and target devices, where the initiator is for initiating and guiding the data exchange process between the parties, target device is the responding device to the initiator.^[^ ^207^ ^]^ Typically, there are two communication modes of NFC: 1) active mode that the devices can generate power supply; 2) passive mode that the initiator generates carrier field and receiving device response by modulating the existing field.^[^ ^206^ ^]^ Recently, NFC has been extensively used in various fields, one popular application is sensor‐enabled NFC tags that can be printed directly on flexible substrates for measuring a multitude of different parameters.^[^ ^208^ ^]^ In particular, flexible substrates that are made from natural bio‐origin materials hold great promise for constructing next‐generation green NFC devices.

For example, Balliu et al. fabricated an NFC tag on paper via selective laser sintering technology.^[^ ^209^ ^]^ The paper substrate allows better absorption of the ink solvent, leading to a smoother surface for NFC tag printing. In addition, Kasuga and coworkers fabricated a degradable device based on wood‐derived nanopaper, which possesses both humidity sensing and wireless information transmission properties.^[^ ^210^ ^]^ The wood‐derived nanopaper with low thermal expansion and thermal resistance allows successful integration of all elements for constructing a nanopaper‐based wireless transmitter. As shown in Figure 10d, the nanopaper sensor device almost decomposes in the soil after 40 days, suggesting great potential for reducing environmental pollution and decreasing the disposal costs of electronics.

## 4 Applications

The flexible electronic devices fabricated with natural bio‐origin materials shows wide applications in different fields due to their biocompatibility with human body and eco‐friendliness with the environment. In this chapter, we discuss the current applications of the flexible electronic devices based on natural bio‐origin materials, which mainly includes three parts: 1) Biomedical implants including passive implants for structural support or implantable electronics for health monitoring that can be absorbed by human body after completing its functions. 2) Artificial e‐skin that can be conformally adhered on human bodies for detecting physical/chemical signals for physiological monitoring;. 3) Environmental monitoring that involves monitoring different kinds of parameters in the ambient environment, constructing sustainable intelligent interfaces between human beings and the environment.

### 4.1 Biomedical Implants

Biomedical implants are extensively employed for diagnosing or treating diverse diseases and clinical issues.^[^ ^14d^ ^]^ The biomedical implants offer specific functions for human body such as biological structure support, controllable drug delivery, and continuous electrophysiological monitoring.^[^ ^211^ ^]^ In contrast to non‐invasive approaches, these implants with better proximity to organs could significantly enhance the sensing accuracy as well as therapeutic effectuality. Regardless of these advantages, the extraordinarily environment inside the human body and the surgeries for implantation impose a limitation on material selection and device fabrication for implantable devices.^[^ ^14c^ ^]^ Therefore, the development of a temporary biomedical implant that can be absorbed by human body after completing its functions is highly desirable.^[^ ^13b^ ^]^ Up till now, various types of bioresorbable materials have been developed as components such as substrates or encapsulation layers for bioresorbable biomedical implants.^[^ ^10^ ^]^ In particular, natural bio‐origin materials have attracted much attention for fabricating biomedical implants due to their large availability and biodegradability.^[^ ^212^ ^]^

Researches on using natural bio‐origin materials for constructing passive implants for structural support have been extensively studied in the past few decades. For passive implants, the used materials are expected to be designed with the following properties: i) adequate incorporation of therapeutics; ii) protect the payloads from damage in vivo while maintaining the bioactivity; iii) release the bioactive agents with predictable profile, and iv) biocompatible and degradable with non‐toxic by‐products. To this end, the natural bio‐origin materials with easy processability, good biocompatibility, and tailorable degradability have been fabricated into different morphology such as films or fibers that can be used as passive implants. For detailed information, readers can check the review articles that summarized the applications of bio‐origin materials such as cellulose,^[^ ^213^ ^]^ chitosan,^[^ ^19^, ^57^, ^214^ ^]^ and SF^[^ ^66^, ^215^ ^]^ in tissue engineering. In addition to passive implants for structural support, natural bio‐origin materials are also widely used in drug delivery devices. Compared with other drug delivery strategies such as oral drug intake, implantable drug delivery devices show excellent delivery efficiency since the drugs can be accurately injected into the target site. Particularly, combined with wireless power transmission technology, many drug delivery devices that offer wirelessly programmable control properties have been proposed. For example, Lee et al.^[^ ^216^ ^]^ developed a device based on oxidized starch integrated with wireless electronic device for drug delivery to brain tumors. The drug molecules are physically trapped and covalently conjugated within the oxidized starch‐based structure. The intracranial drug delivery process can be controlled by mild‐thermic actuation. The entire device can be biodegraded, which provides biocompatibility and minimizes potential neurological side‐effects. This work indicates that the wirelessly controlled, bioresorbable drug delivery devices hold great promise for in‐situ diagnosis and on‐demand therapy.

**Figure 11:**
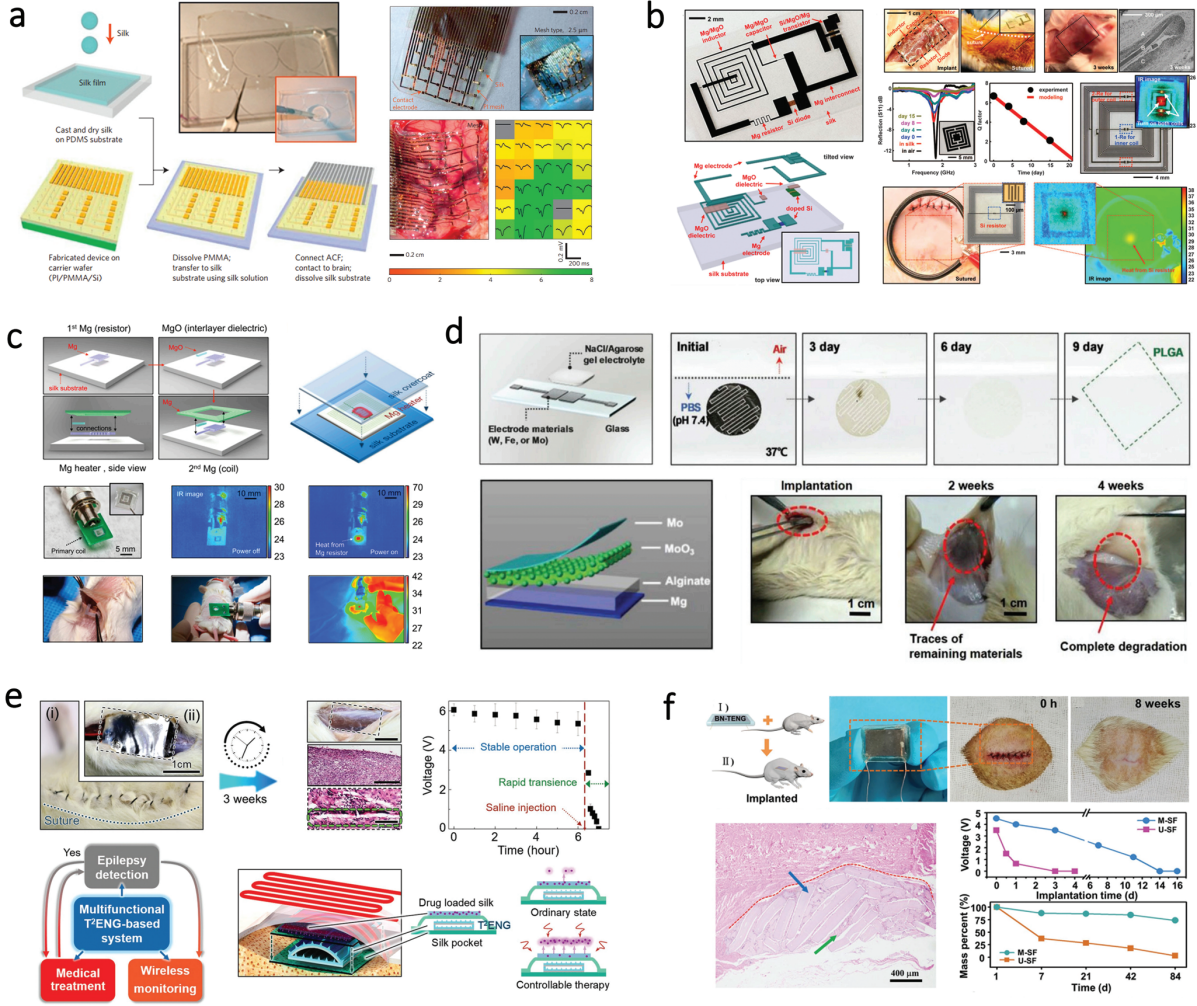
Biomedical implants based on different kinds of bio‐origin materials. a) Ultrathin conformal bio‐integrated electronics based on SF for implantable and surgical applications. Reproduced with permission.^[^ ^218^ ^]^ Copyright 2010, Springer Nature. b) A transient bioresorbable device based on silk substrate for thermal therapy. Reproduced with permission.^[^ ^219^ ^]^ Copyright 2012, American Association for the Advancement of Science. c) Silk‐based resorbable electronic devices for remotely controlled therapy and in vivo infection abatement. Reproduced with permission.^[^ ^215d^ ^]^ Copyright 2014, National Academy of Sciences, USA. d) A fully biodegradable Mg‐MoO_3_ battery for in vivo on‐board power supply. Reproduced with permission.^[^ ^220^ ^]^ Copyright 2018, Wiley‐VCH. e) Silk‐based implantable and biodegradable self‐powered systems for real‐time in vivo monitoring and therapeutic treatments of epileptic seizures. Reproduced with permission.^[^ ^221^ ^]^ Copyright 2018, Wiley‐VCH. f) Fully bioabsorbable TENG based on natural materials for in vivo power supply. Reproduced with permission.^[^ ^115^ ^]^ Copyright 2018, Wiley‐VCH.

In addition, bioresorbable “active implants” including sensors, energy devices, and actuators for medical requirements have also been developed recently.^[^ ^217^ ^]^ To construct bioresorbable and implantable integrated electronic systems, the components of the electronic devices should be bioresorbable. The natural bio‐origin materials with excellent biodegradability usually serve as the temporary, soluble supporting substrate for the implantable electronic devices. Particularly, silk is the most extensively used material due to its biocompatibility and controllable water solubility. For example, Kim et al. designed a bio‐system supported by bioresorbable SF substrates.^[^ ^218^ ^]^ As shown in **Figure** **11**a, when the device is attached to tissue, the SF substrate would be dissolved, initiating an autonomous and conformal wrapping process via capillary force at the interface. In addition, the ultrathin electronic is designed with specialized mesh, which ensures the high conformality between the device and the curvilinear surfaces of the tissue. To further developed an implantable integrated electronic system, Hwang and coworkers proposed an implantable transient device with the integration of sensor, actuator, power supply system, and wireless control strategy, as shown in Figure 11b.^[^ ^219^ ^]^ Silk is utilized as the substrate and packaging material due to its water solubility and enzymatical degradability. The fabricated device is then implanted in the subdermal area of BALB/c mice. Results show that after 3 weeks, only slight residues can be observed, with evidence of slow reintegration into the subdermal layers, which demonstrate that this device can further provide opportunities for biomedical applications. Tao et al. developed a degradable therapeutic device base on silk substrate, which can be wirelessly activated to provide necessary medical treatment after implantation.^[^ ^215d^ ^]^ By entraining and stabilizing drugs within the silk material matrix, the devices can act as wireless drug delivery systems to provide programmable remote control for drug releasing. As shown in Figure 11c, the device can be resorbed into the surrounding tissue without causing any side effects, thus eliminating the need for retrieval. Furthermore, the lifetime of these implants can be tunable by using silk as the encapsulating material, leading to controllable dissolution rates of devices ranging from minutes to weeks.

The power supply device also plays a vital role in implantable electronics to ensure their long‐term function.^[^ ^222^ ^]^ Ideally, an implantable power device is expected to have the following features: miniaturized, high affinity with biological soft tissues, and fully bioresorbable. So far, different kinds of power devices including power generation and energy storage devices have been developed.^[^ ^223^ ^]^ The advancement of natural bio‐origin materials that vague the interface between devices and tissues can enhance their adhesion to tissues. For instance, Huang et al. fabricated a bioresorbable magnesium‐molybdenum oxide (Mg‐MoO_3_) battery based on calcium crosslinked alginate as the electrolyte, as shown in Figure 11d.^[^ ^220^ ^]^ MoO_3_ is a suitable cathode material for fabricating biodegradable batteries due to its solubility in aqueous solution and desirable biocompatibility at a controlled level. Results show that an output voltage about 1.6 V of a single cell battery can be obtained, which can be used for powering the amplifier of an electrocardiogram (ECG) signal detector. Besides, in vivo test using a rat model demonstrate that the battery can be fully biodegraded within 4 weeks, without causing obvious inflammatory effects. In addition to biodegradable battery, other power device such as TENGs have also been widely applied to implantable applications. For instance, Zhang et al. reported a TENG using longitudinally patterned Mg and silk.^[^ ^221^ ^]^ Here, silk is used as both encapsulation and triboelectric material. As shown in Figure 11e, in vivo experiments are performed on TENG implanted in the subdermal region of mice. The implanted TENG shows an output voltage of 6 V for 6 h under constantly applied external force. Then, degradation of the TENG can be triggered by injecting physiological saline solution at the implant region. After three weeks, the implant region exhibits gradual reintegration and apparent revascularization without apparent inflammatory reactions, demonstrating excellent in vivo biocompatibility of the TENG. In addition, Jiang et al. fabricated fully biodegradable TENGs based on five different natural bio‐origin materials that are biodegradable and easily processable.^[^ ^115^ ^]^ As illustrated in Figure 11f, the biocompatibility of these materials is tested using L929 cells with hematoxylin and eosin stained histologic section. After being implanted, the life time of the TENG is controllable via different SF encapsulation.

### 4.2 Artificial e‐Skin

Artificial e‐skin that can be directly adhered on human bodies for measuring physical signals have attracted great attention in virtue of their applications in human motion detection as well as human‐machine interaction with high efficiency as well as minimum discomfort.^[^ ^224^ ^]^ To ensure the high affinity of the artificial e‐skin with a human body, mechanical flexibility is one of the most important features. Specifically, these e‐skin mounted directly onto human bodies are expected to collect different physical signals (e.g., humidity, pressure, or temperature) and convert such stimuli into visualized information.^[^ ^182^, ^189^ ^]^ Typically, synthetic flexible polymers such as PET, PDMS, and PI combined with functional sensing materials are commonly used for the construction of artificial e‐skin. However, these synthetic polymers are usually non‐degradable, which would give rise to a large amount of E‐waste. Besides, some polymer‐based substrates are neither biocompatible nor air permeable, which may cause damage to the human body.^[^ ^225^ ^]^ Natural bio‐origin materials such as cellulose, chitosan, gelatin, collagen, and silk provides promising platforms for artificial e‐skin because of their biodegradability, remarkable mechanical toughness, and large availability.^[^ ^67^, ^226^ ^]^ Besides, other advantages such as hierarchical structure and morphological diversity also makes them as promising building blocks for fabricating artificial e‐skin.^[^ ^17^, ^20^, ^65^, ^227^ ^]^

**Figure 12:**
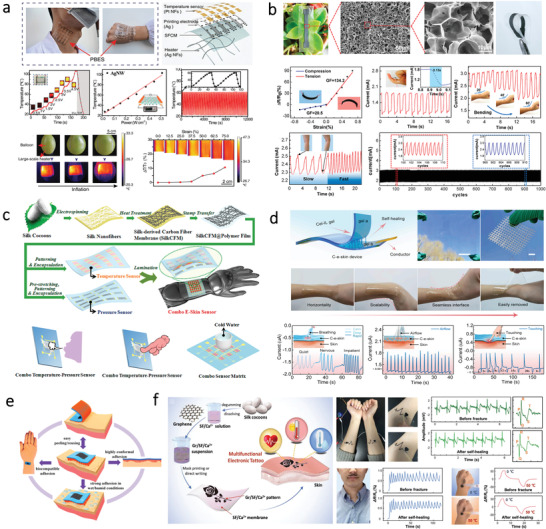
Artificial e‐skin based on different kinds of bio‐origin materials. a) Silk protein‐based device for temperature sensing of human body. Reproduced with permission.^[^ ^228^ ^]^ Copyright 2020, Wiley‐VCH. b) Flexible sensor based on starch film for monitoring human motions. Reproduced with permission.^[^ ^229^ ^]^ Copyright 2019, American Chemical Society. c) Silk‐derived e‑skin for temperature and pressure sensing. Reproduced with permission.^[^ ^230^ ^]^ Copyright 2017, American Chemical Society. d) Biomimetic e‐skin based on cellulose ionic hydrogel for sensing object contact, air flow, and temperature. Reproduced with permission.^[^ ^231^ ^]^ Copyright 2019, Elsevier. e) Microstructure silk fibroin adhesives for flexible skin sensors. Reproduced with permission.^[^ ^232^ ^]^ Copyright 2020, American Chemical Society. f) Self‐healable multifunctional e‐tattoos based on SF. Reproduced with permission.^[^ ^233^ ^]^ Copyright 2019, Wiley‐VCH.

So far, different kinds of artificial e‐skin based on natural bio‐origin materials have been developed for measuring physical signals such as pressure, humidity, or temperature of the human body. For instance, Huang et al. designed an SF composite membrane that could be used for temperature monitoring.^[^ ^228^ ^]^ As shown in **Figure** **12**a, the composite membrane is fabricated via mesoscopic doping of regenerated SF. The composite membrane is also inflammation‐free and air‐permeable, which can be directly attached to human skin for continuous thermal management. Apart from single signal monitoring, researchers also fabricated a multimodal artificial e‐skin that can detect two or more stimuli. For instance, Liu and coworkers fabricated a multifunctional and degradable sensor via integrating laser‐induced porous carbon with a starch film.^[^ ^229^ ^]^ The obtained sensor can be used for strain, temperature, and pressure detection (Figure 12b). The sensor can be dissolved in water, indicating its degradability without causing any contamination.

Specifically, to obtain accurate signals from the human body, multifunctional e‐skin that can sense diverse stimuli such as temperature, pressure, and humidity has been extensively studied. Wang et al. reported a temperature‐pressure e‐skin using carbon fiber membranes derived from transparent SF as the active sensing material.^[^ ^230^ ^]^ As shown in Figure 12c, the obtained e‐skin can achieve multiple functions including detecting exhaling, sensing finger‐press, and measuring temperature distribution. In addition to multiple stimuli sensing, the e‐skin is also expected to have self‐healing ability for mimicking real human skin. Zhao et al. fabricated a self‐healable e‐skin using cellulose ionic hydrogel (Cel‐IL dynamic gel), as shown in Figure 12d.^[^ ^231^ ^]^ The Cel‐IL dynamic gel displays tunable mechanical properties and self‐healing ability due to its tunable dynamic hydrogen bonding and reversible microstructures under different humidity level, which holds great promise in various applications. The e‐skin constructed from this Cel‐IL dynamic gel can discriminate various mechanical strains, finger touch, and airflow signals with high sensitivity. Liu and coworkers fabricated a SF‐based adhesive with a micropillar structure for obtaining conformal and biocompatible adhesion with skin surface.^[^ ^232^ ^]^ As illustrated in Figure 12e, the obtained adhesive displays reliable and stable bonding with skin surface. In addition, the adhesive can also be peeled off from skin easily without any hurting, demonstrating its great potentials to serve as a reliable adhesive for conformal sensors.

To further enhance the conformal contact between skin and conformal sensor, lightweight and ultra‐soft electronic tattoos (e‐tattoos) that can be intimately attached onto human skin have been developed recently. Wang et al. fabricated an e‐tattoo by integrating graphene with skin‐like soft SF/Ca^2+^ films (Gr/SF/Ca^2+^), which can simultaneously detect strain, humidity, and temperature of the human body.^[^ ^233^ ^]^ As shown in Figure 12f, the soft e‐tattoo with skin‐like flexibility can be intimately attached to human skin without mechanical failure under skin deformation, which can sensitively monitor various sensations such as ECG, respiration, and temperature.

### 4.3 Environmental Monitoring

Environmental monitoring is a broad concept that involves monitoring different kinds of parameters in the ambient environment such as humidity level,^[^ ^234^ ^]^ temperature,^[^ ^235^ ^]^ volatile organic compounds (VOC),^[^ ^236^ ^]^ and so on. It requires various sensors that can convert physical or chemical stimuli into measurable signal as well as energy devices for power supply. Particularly, to eliminate the E‐waste caused by these electronic devices to the environment, natural bio‐origin materials are thus extensively used for fabricating electronics that are environmentally‐friendly while maintaining their functions.

**Figure 13:**
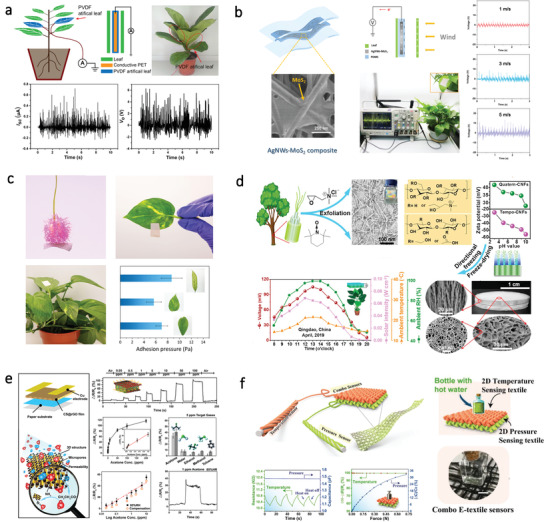
Environmental monitoring applications. a) Leaf‐based TENG for self‐powered wind speed monitoring. Reproduced with permission.^[^ ^237^ ^]^ Copyright 2019, Elsevier. b) A stretchable PDMS‐based TENG attached to plant leaf for self‐powered wind speed monitoring. Reproduced with permission.^[^ ^105^ ^]^ Copyright 2019, Elsevier. c) A breathable TENG self‐attached on plant for environmental energy harvesting. Reproduced with permission.^[^ ^238^ ^]^ Copyright 2021, American Chemical Society. d) CNF‐based aerogel for plant transpiration monitoring. Reproduced with permission.^[^ ^239^ ^]^ Copyright 2020, Elsevier. e) Chitosan‐based chemical sensor for VOCs detection. Reproduced with permission.^[^ ^240^ ^]^ Copyright 2017, The Royal Society of Chemistry. f) Multifunctional sensor for temperature and pressure sensing. Reproduced with permission.^[^ ^241^ ^]^ Copyright 2019, Wiley‐VCH.

For instance, Feng et al. fabricated a leaf‐based TENG with environmentally degradable leaves as the triboelectric material for harvesting wind energy from nature.^[^ ^237^ ^]^ As shown in **Figure** **13**a, an artificial tree is fabricated to simulate wind energy harvesting process in the ambient environment. Results show that output voltage and current can be generated when the artificial tree is blown, indicating the potential of this TENG in energy harvesting and self‐powered sensors for wind speed monitoring. Besides, as a natural material, leaf can also serve as a frictional positive layer for single‐electrode TENG. Our group developed a plant‐supported stretchable TENG based on AgNWs‐MoS_2_ composite film and PDMS.^[^ ^105^ ^]^ As show in Figure 13b, when attached to a plant leave, the stretchable TENG could serve as a self‐powered wind speed sensor. However, the PDMS‐based device exhibits low breathability, which may influence normal plant respiration and is not allowed for long‐term on‐plant usage. Therefore, to improve the biocompatibility of the device, we further developed a waterproof and breathable TENG by means of the electrospinning and electrospraying method.^[^ ^238^ ^]^ As shown in Figure 13c, the TENG attached to a plant leaf can not only harvest natural energy but also serve as a self‐powered wind speed sensor.

In addition to wind speed, the humidity level is also an important parameter. Yang and coworkers developed an asymmetric ionic aerogel based on oppositely‐charged nanofibrils by freeze‐casting technique, which can generate electricity from moisture.^[^ ^239^ ^]^ As shown in Figure 13d, the fabricated aerogel would be hydrated when exposed to moisture, then the dissociation and diffusion ions can induce directional charge movement, thereby generating voltage. Results show this biological aerogel could generate different voltage under different humidity levels, indicating its usage as a self‐powered humidity sensor. Being installed near a plant, the biological aerogel can be used for plant transpiration monitoring, suggesting its potential applications in agricultural field.

VOCs are toxic air pollutants, so the accurate monitoring of VOCs has attracted intensive attention for safety controls of the environment. Wang et al. reported a flexible chemical sensor with a 3D biomimetic structure constructed by chitosan and GO (CS@GO).^[^ ^240^ ^]^ As shown in Figure 13e, by depositing the CS@rGO on the cellulose paper, a flexible chemical sensor can be assembled, which displays high‐performance detection of VOCs, including fast response time and low detection limit. In addition, multifunctional sensing is also important in environmental monitoring. Wu et al. developed a strategy for the fabrication of flexible and multifunctional electronic textiles using functionalized silkworm fiber coiled yarns.^[^ ^241^ ^]^ As illustrated in Figure 13f, a combined sensor array can be fabricated by sewing the temperature and pressure sensing yarns together. This combo sensor is capable of sensing temperature and pressure separately with position precision, showing great potential in multidimensional sensing applications.

## 5 Conclusion and Future Perspectives

In conclusion, the advantages of bio‐origin materials such as abundant availability, biocompatibility, programmable biodegradability, and environmental benignity make them promising for constructing eco‐friendly flexible devices. Besides, due to the remarkable development in processing technology and the diverse morphologies of bio‐origin materials, researchers have explored multiple ways to fabricate functional materials with unique structures and desirable performances. These advantages render them promising alternatives for conventional inorganic materials or non‐biodegradable synthetic polymers for developing green flexible electronics. The past decades have witnessed the fast‐growing advancements of various bio‐origin material‐based flexible devices ranging from fields of energy management to information communication. Furthermore, the degradation behaviors of bio‐origin materials allow the construction of bioresorbable electronics, showing great potential applications in biomedical implants. In addition, other versatile applications such as artificial e‐skin and environmental monitoring can also be realized. Regardless of the important advancement of bio‐origin material‐based flexible devices, some problems are still need to be addressed.

From the materials' point of view, massive production of bio‐origin materials remains complex and high energy consumption. Given the relatively complicated process for producing bio‐origin materials, developing novel and cost‐effective manufacturing technologies with large scalability is highly desirable. In addition, although the bio‐origin materials can be made into flexible substrates or dielectric/triboelectric layers for flexible devices, other essential functional compositions such as metals or conductive polymers would pose limitations to the total biocompatibility of the devices. Therefore, further researches are required to endow the bio‐origin materials with concrete properties such as electrical conductivity, electrochemical activity as well as chemical features, which may be realized by chemical modification, element substitution, and interfacial structure design.

From the device point of view, although numerous devices based on bio‐origin materials have been fabricated, applications of these devices are still limited in academic research. To fabricate bio‐origin material‐based flexible devices for industrial production is a possible future research direction. For energy storage devices, designing thick electrodes with high mass loading or pore manufacture is highly desirable for enhancing energy density of the devices. Another challenge is the power supply for implantable devices. So far, conventional battery is commonly used for powering the implantable devices. However, a surgical operation is required for periodical change of the battery. Although piezoelectric or triboelectric nanogenerators have already been used for in vivo applications, they are improbable to provide enough and sustained energy to power devices. In this case, developing a rechargeable battery that can be integrated with energy harvesters is a possible solution for providing sufficient and continuous power supply. Currently, it is still quite challenging to propose a general solution for addressing all the power supply problems of implanted devices based on natural bio‐origin materials, advancements that integrate various power options offer multifunctional platforms for diverse and facile biomedical systems. As such, future efforts are expected to focus on developing advanced integration techniques such as surface or interface engineering technology to construct new sustainable systems that integrates diverse devices with different functions, such as power generation, energy storage, and sensing capabilities.

For the applications, flexible devices based on bio‐origin materials have been extensively used in different fields such as biomedical implants, human motion detection, and so forth. However, there are few publications about the use of bio‐origin material‐based devices in horticulture or agriculture, which can be ascribed to the fact that flexible electronics are generally uncommon in this field. Whereases, there is a fast‐growing trend for utilizing flexible electronics for agricultural information sensing by intelligently interfacing plants with flexible sensors, which allows monitoring the surrounding microclimates and the physiological information of plants.^[^ ^242^ ^]^ However, it remains a challenging task for multiple sensing simultaneously due to the relatively complex signaling pathways in plants. Moreover, for an intelligent agriculture system, it is troublesome to retrieve all the sensing devices installed among the cultivated field, which means that the uncollected sensing devices may induce pollution to the environment or the farmland. As such, fabricating on‐spot and degradable/recyclable sensing devices based on natural bio‐origin materials is a promising future research direction for building an intelligent and sustainable agriculture system.

Based on natural bio‐origin materials, we desire to present a roadmap for sustainable development of future flexible devices, achieving a good balance between the intrinsic advantages of natural materials and reliable performances of the “green” electronics. This concept would lead the development of flexible devices from traditional mountable device to emerging adhesive device, as well as the desirable on‐spot devices, reducing the concerns of environment pollution and narrowing the technology gap for future human‐machine‐environment interactions.

## Conflict of Interest

The authors declare no conflict of interest.
